# Efficacy and safety of low-dose prasugrel as dual antiplatelet therapy in patients with ischemic heart disease: a systematic review and network meta-analysis of randomized controlled trials

**DOI:** 10.1007/s12928-025-01129-2

**Published:** 2025-06-09

**Authors:** Toshiharu Fujii, Kazushige Amano, Satoshi Kasai, Yota Kawamura, Fuminobu Yoshimachi, Yuji Ikari

**Affiliations:** 1 Department of Cardiovascular Medicine, Tokai University School of Medicine, 143 Shimokasuya, Isehara, 259-1193 Japan; 2 Department of Cardiovascular Medicine, Tokai University Hachioji Hospital, Hachioji, Japan

**Keywords:** Dual antiplatelet therapy, Major adverse cardiovascular event, Bleeding event, Prasugrel, Low dose, Network meta-analysis

## Abstract

Low-dose prasugrel could provide a better balance between adverse ischemic and bleeding events compared to other P2Y12 receptor inhibitors as part of dual antiplatelet therapy (DAPT) for patients with ischemic heart disease. This study evaluated these risks of adverse events associated with low-dose prasugrel and other P2Y12 receptor inhibitors. A network meta-analysis was conducted, searching for randomized controlled trials (RCTs) comparing clopidogrel (75 mg), low-dose (3.75 mg) and standard-dose (10 mg or 5 mg) prasugrel, or ticagrelor (180 mg). The primary endpoint was major adverse cardiovascular events (MACE), a composite of cardiovascular death, myocardial infarction, or stroke. The secondary endpoint was major bleeding, cardiovascular death, myocardial infarction, and stroke. Across 13 RCTs, neither low-dose prasugrel, standard-dose prasugrel, nor ticagrelor showed a statistically significant difference in MACE compared to clopidogrel [risk ratio (RR): 0.73, 95% confidence interval (CI) 0.49–1.09; RR: 0.86, 95% CI 0.68–1.09; RR: 1.02, 95% CI 0.62–1.67, respectively]. However, the standard dose of prasugrel was associated with a significantly higher risk of bleeding events compared to clopidogrel (RR, 0.72; 95% CI 0.35–1.49; RR, 1.26; 95% CI 1.01–1.58; RR, 1.26; 95% CI 0.82–1.96). The surface under the cumulative ranking curves was highest for low-dose prasugrel for both MACE and bleeding events (17.3 and 64.6 for clopidogrel, 84.5 and 84.9 for low-dose prasugrel, 62.0 and 11.8 for standard-dose prasugrel, and 36.2 and 38.7 for ticagrelor, respectively). Low-dose prasugrel may be a viable option in addition to standard P2Y12 receptor inhibitors.

**Graphical abstract:**

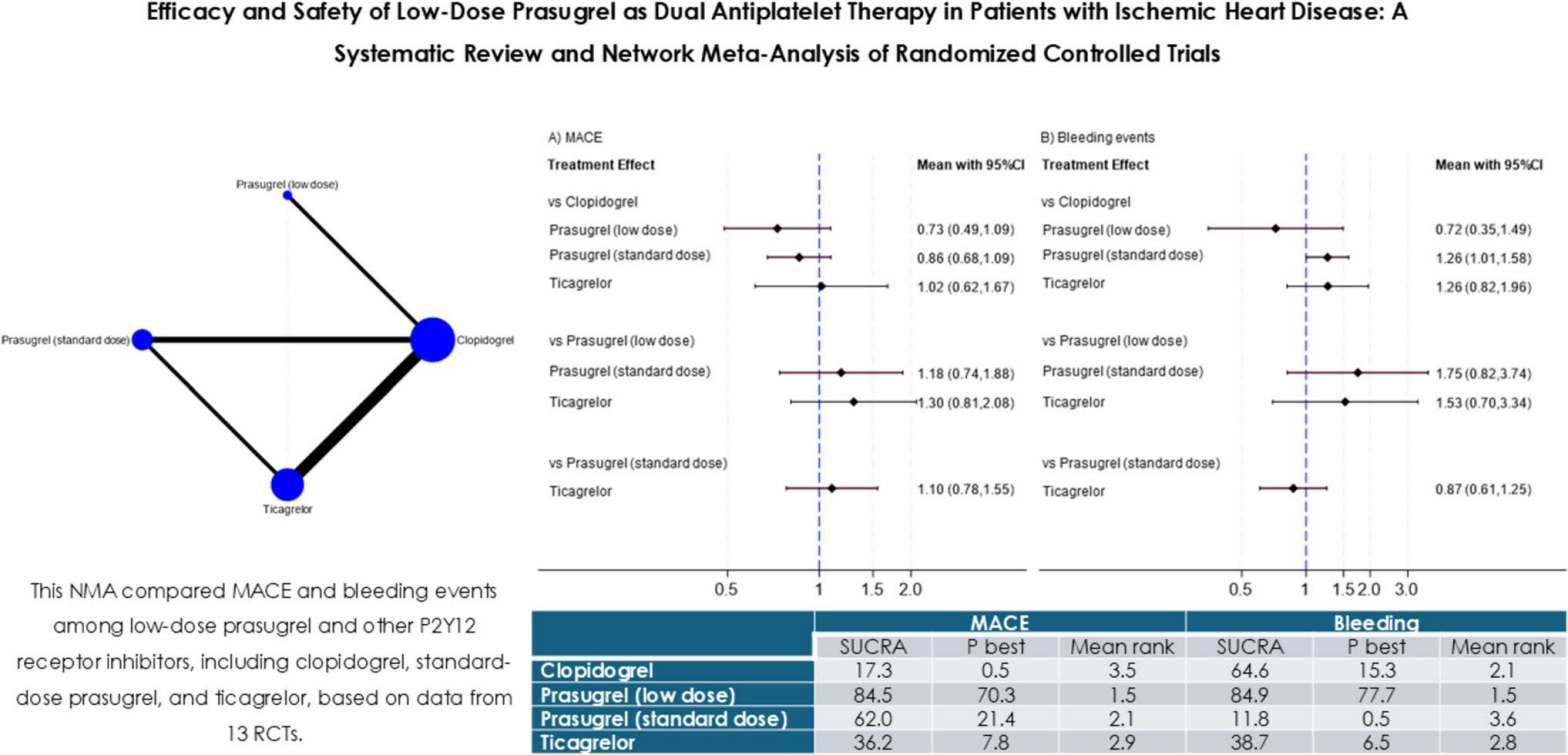

**Supplementary Information:**

The online version contains supplementary material available at 10.1007/s12928-025-01129-2.

## Introduction

Dual antiplatelet therapy (DAPT) has been the established regimen for patients with ischemic heart disease who have undergone recent stent implantation. Initially, ticlopidine, a first-generation P2Y12 receptor inhibitor, was used to prevent stent thrombosis after bare metal stent implantation. However, clopidogrel was developed to enhance effectiveness and safety, ultimately replacing ticlopidine. Currently, clinicians have the option to choose from three types of P2Y12 receptor inhibitors: clopidogrel, prasugrel, and ticagrelor.

From the perspective that the primary goal of P2Y12 receptor inhibitors is to reduce ischemic events while minimizing contradictory bleeding events, the balance between them has been compared in numerous large-scale studies. In recent years, options such as prasugrel and ticagrelor have been preferred over conventional clopidogrel [1].

Several randomized controlled trials (RCTs) have suggested a higher bleeding risk with prasugrel than with clopidogrel, which has raised concerns about the optimal dosage of P2Y12 receptor inhibitors. Low-dose prasugrel has been proposed as a potential option to improve the balance of adverse events, with several studies suggesting potential better outcomes.

However, the relative position of low-dose prasugrel compared to other P2Y12 receptor inhibitors remains unclear due to limited reports on its effectiveness and safety.

This systematic review conducted a network meta-analysis (NMA) to determine where low-dose prasugrel stands compared to other P2Y12 receptor inhibitors as part of DAPT for ischemic heart disease.

## Methods

### Data availability statement

The authors declare that the supporting data used in this NMA are available.

### Study design, data sources, and search

The completed review has been prepared following the Preferred Reporting Items for Systematic Reviews and Meta-analyses for Network Meta-Analyses (PRISMA-NMA, Supplemental Table 1) [2].

This NMA aimed to investigate the efficacy and safety of P2Y12 receptor inhibitors in patients with ischemic heart disease. Three P2Y12 receptor inhibitors, as part of DAPT in combination with aspirin, were compared: clopidogrel (75 mg maintenance dose), standard-dose prasugrel (10 mg or adjusted 5 mg based on age or body weight), low-dose prasugrel (3.75 mg maintenance dose), and ticagrelor (90 mg twice a day maintenance dose). Due to the inclusion of component trials in this NWA and the availability of publicly accessible data, this review—constituting a secondary publication utilizing previously published trials—was exempt from institutional review board approval.

Two independent investigators (TF and KA) performed the literature search using Ovid Medline, Scopus, and the Cochrane Central Register of Controlled Trials (CENTRAL) databases published between 1998 and November 2023. The full search strategy is outlined in Supplemental Table 2a–c. The main inclusion criteria were (1) RCTs investigating oral P2Y12 receptor inhibitors (clopidogrel, prasugrel, or ticagrelor) as part of DAPT administered with aspirin in patients with ischemic heart disease, (2) studies designed to investigate clinical outcomes, (3) studies reporting major adverse cardiovascular events and bleeding events, and (4) studies with a follow-up period beyond 3 months. Studies were excluded if they (1) compared only different regimens of the same P2Y12 receptor inhibitor, (2) included anticoagulation therapy (3) were primarily designed for pharmacokinetics/pharmacodynamics analysis of platelet reactivity, (4) were randomized based on genotype, (5) had a crossover design, or (6) had a nonrandomized design. The two investigators (SK and YK) independently screened articles first by title and abstract after removing duplicates, and then by full text based on pre-specified criteria. Any disagreements were resolved by a third investigator (YI).

### Quality assessment

Among the RCTs that met the eligibility criteria, the risk of bias (ROB) was independently assessed by two investigators (TF, FY) using Version 2 of the Cochrane risk-of-bias tool for randomized trials (RoB 2); 5 domains of bias were evaluated: (1) randomization process, (2) deviations from intended interventions, (3) missing outcome data, (4) measurement of the outcome, and (5) selection of reported results [3] The certainty of evidence for each outcome was evaluated using the GRADE approach [4].

### Study outcomes

The primary efficacy endpoint was major adverse cardiovascular events (MACE), defined as a composite of cardiovascular death, nonfatal myocardial infarction (MI), or stroke. The secondary safety endpoint was major bleeding, defined according to (1) Thrombolysis In Myocardial Infarction (TIMI) bleeding criteria [5], (2) Bleeding Academic Research Consortium (BARC) criteria [6], and (3) Platelet Inhibition and Patient Outcomes (PLATO) bleeding criteria. [7] Definitions of these outcomes are summarized in Supplemental Table 3a–e.

### Statistical analysis

After the assumption of homogeneity of the included studies and transitivity was established, we used a random-effects model to pool the effect sizes. Outcomes were reported as risk ratios (RRs) with corresponding 95% confidence intervals (CIs) [8, 9]. Clopidogrel was selected as the reference treatment in this NMA due to its frequent use as a control arm in many RCTs and its suitability as an anchor for analysis. The DerSimonian and Laird random-effects model was used for direct pairwise comparison meta-analyses to report direct estimates. Global inconsistency was assessed using the inconsistency model, specifically the design-by-treatment interaction model of Higgins et al [10]. Local inconsistency within loops formed by two or more studies was assessed using the node-splitting model of Dias et al [11, 12]. This confirmed whether estimated effects from direct comparisons were consistent with those from indirect comparisons. *P* > 0.05 indicated no significant difference in estimated effects between direct and indirect comparisons [13]. Network heterogeneity across all treatment contrasts was assessed using Higgins and Thompson’s *I*^2^ statistics, Cochrane’s Q statistic, and associated *P* values (*P* < 0.05 considered significant) [14]. To rank treatments for each MACE and bleeding event, we used the surface under the cumulative ranking curve (SUCRA) probabilities [15]. SUCRA values, expressed as percentages, compare each intervention to an imaginary intervention that is always the best without uncertainty.

Potential publication bias was visually assessed using funnel plots, and small-study bias was evaluated at the outcome level using Egger’s test.

The study conducted a pre-specified sensitivity analysis by excluding studies meeting the following criteria: (1) fewer than 200 cases in one treatment arm and (2) exhibiting high RoB. Sensitivity analysis to test the stability of statistical results was performed by omitting each study iteratively and recalculating pooled RRs and 95% CIs for the remaining studies.

All statistical calculations were performed using STATA statistical software version 18.0 (Stata Corporation, College Station, TX, USA) and R 4.40 (The R Project for Statistical Computing, Vienna, Austria).

## Results

### Search results and study characteristics

**Fig. 1:**
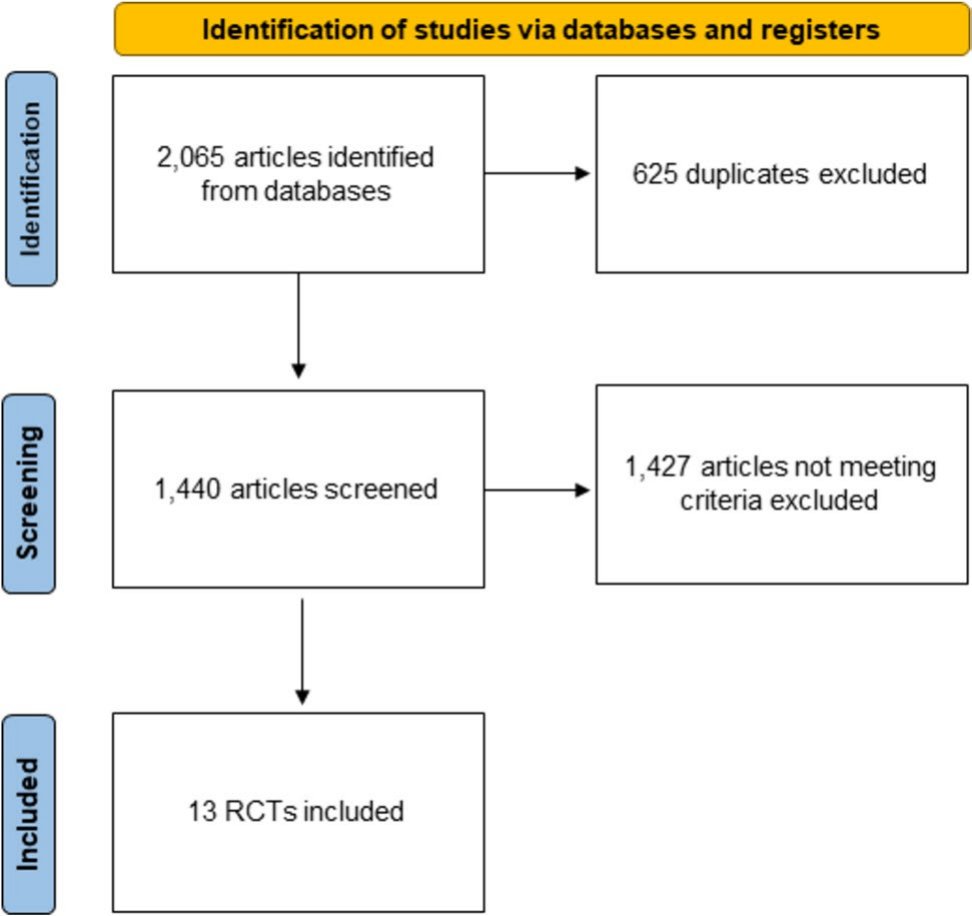
PRISMA flow diagram. PRISMA 2020 flow diagram for new systematic reviews that included searches of databases and registers only

After initially identifying articles meeting the selection protocol, 1440 articles were screened following the removal of duplicates. Of these, 1427 articles were excluded based on title and abstract, and finally, 13 full-text articles comprising 53,216 patients were included for analysis (Fig. 1). In the DISPERSE-2 Trial, a 3-arm study involving 75 mg of clopidogrel and 180 mg and 360 mg of ticagrelor, the 360-mg ticagrelor arm was excluded from the present NWM because it is no longer clinically utilized [16]. Consequently, the trial was treated as a 2-arm study with 75 mg of clopidogrel and 180 mg of ticagrelor.

**Fig. 2:**
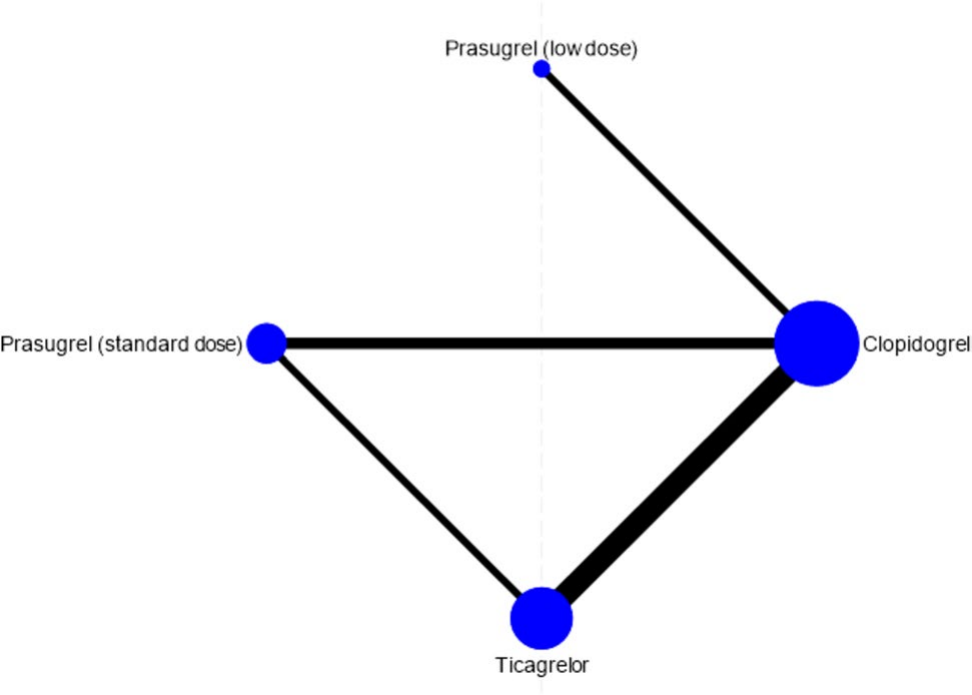
Network of treatment comparisons. Nodes represent antiplatelet agents, and a directly connected line indicates that a direct comparison between the two drugs was reported. The size of the nodes and the width of the lines reflect the number of studies

Table 1 shows the baseline demographic characteristics of patients in the included trials. The 13 trials included two comparing clopidogrel and prasugrel (low dose) (PRASFIT-ACS and PRASFIT-Elective) [17, 18], three trials comparing clopidogrel and prasugrel (standard dose) (Elderly ACS 2, TRILOGY ACS, and TRITON-TIMI 38) [19–21], six trials comparing clopidogrel and ticagrelor (DISPERSE-2, PHILO, PLATO, TICAKORIA, Tang et al., and Wang et al.) [7, 16, 22–25], and two trials comparing prasugrel (standard dose) and ticagrelor (ISAR REACT 5 and PRAGUE 18) (Supplemental Table 4a–c) [26, 27]. Network evidence for comparisons between the antiplatelet agents is shown in Fig. 2.

**Table 1:** Summary of included trials

Trial	Enrollment	Intervention, no. of patients	Efficacy outcome	Safety outcome	Follow-up	Study setting
TICAKORIA Park et al. [23]	2014–2017	T 90 mg, 400 C 75 mg, 400	MACE defined as a composite of death from cardiovascular causes, nonfatal MI, or nonfatal stroke)	A composite of major bleeding or minor bleeding according to the PLATO criteria	12 months	ACS with planned invasive management
ISAR REACT 5 Schupke et al. [26]	2013–2018	T 90 mg, 2012 P 5/10 mg, 2006	The composite of death, MI, or stroke	Bleeding defined as type 3, 4, or 5 on the BARC scale	12 months	ACS with planned invasive evaluation
Elderly ACS 2 Savonitto et al. [19]	2012–2017	P 5 mg, 713 C 75 mg, 730	The composite of all-cause mortality, MI, disabling stroke, and rehospitalization for cardiovascular causes or bleeding	BARC type 2 or 3	12 months	Elderly (aged > 74 years) ACS treated with PCI
PRAGUE 18 Motovska et al. [27]	2013–2016	P 5/10 mg, 634 T 90 mg, 596	The occurrence of cardiovascular death, nonfatal MI, or stroke	Bleeding defined according to the TIMI and BARC criteria	12 months	AMI treated with primary PCI
Tang et al. [24]	2013–2015	T 90 mg, 200 C 75 mg, 200	The composite of overall death, MI, unplanned revascularization, and stroke	Bleeding defined by TIMI criteria	6 months	STEMI treated with primary PCI
PHILO Goto et al. [22]	NA	T 90 mg, 401* C 75 mg, 400*	The composite of death from vascular causes, MI, or stroke	Major bleeding defined by PLATO criteria	12 months	ACS with planned PCI
PRASFIT-Elective Isshiki et al. [18]	2011–2012	P 3.75 mg, 370 C 75 mg, 372	The composite of cardiovascular death, nonfatal MI, and nonfatal ischemic stroke	Non-CABG-related bleeding defined by TIMI criteria	24 weeks	Stable angina or prior MI treated with elective PCI
PRASFIT-ACS Saito et al. [17]	2010–2012	P 3.75 mg, 685 C 75 mg, 678	The composite of cardiovascular death, nonfatal MI, and nonfatal ischemic stroke	Non-CABG-related bleeding defined by TIMI criteria	24 weeks	ACS treated with PCI
TRILOGY ACS Roe et al. [20]	2008–2011	P 5/10 mg, 4663 C 75 mg, 4663	The composite of death from cardiovascular causes, nonfatal MI, or nonfatal ischemic stroke	Bleeding is defined as severe or life-threatening according to GUSTO criteria, and as major according to TIMI criteria, not related to CABG	30 months	ACS without revascularization
PLATO Wallentin et al. [7]	2006–2008	T 90 mg, 9333 C 75 mg, 9291	The composite of death from vascular causes, MI, or stroke	Major bleeding defined by PLATO criteria	12 months	ACS
Wang et al. [25]	2013–2014	T 90 mg, 100 C 75 mg, 100	The composite of MI, stroke, and cardiovascular death	PLATO major bleeding, and PLATO minor bleeding	12 months	Elderly (aged ≥ 65 years) ACS
TRITON-TIMI 38 Wiviott et al. [21]	2004–2007	P 10 mg, 6813 C 75 mg, 6795	The composite of death from cardiovascular causes, nonfatal MI, or nonfatal stroke	TIMI major bleeding not related to CABG, non-CABG-related TIMI life-threatening bleeding, and TIMI major or minor bleeding	15 months	ACS treated with scheduled PCI
DISPERSE-2 Cristopher et al. 2007	2004–2005	T 180 mg, 323 T 90 mg, 334 C 75 mg, 327	Individual and composite incidence of MI (including silent MI), death, stroke, and severe recurrent ischemia	Protocol-defined major or minor bleeding	12 weeks	NSTE-ACS

*C* clopidogrel, *P* prasugrel, *T* ticagrelor, *MACE* major adverse cardiovascular event, *PLATO* platelet inhibition and patient outcome, *ACS* acute coronary syndrome, *MI* myocardial infarction, *BARC* bleeding academic research consortium, *PCI* percutaneous coronary syndrome, *TIMI* thrombolysis in myocardial infarction, *STEMI* ST-elevation myocardial infarction, *CABG* coronary artery bypass grafting, *GUSTO* global use of strategies to open occluded coronary arteries, *NSTE-ACS* non-ST-elevation acute coronary syndrome

*At the safety endpoint, 387 patients in ticagrelor and 380 patients in prasugrel

### Risk of bias

The trials were evaluated for RoB using ROB 2 and categorized as shown in Supplemental Figure 1a and 1b. Five trials were classified as “low risk” (38.5%), five trials as “some concerns” (38.5%), and three trials as “high risk” (23.1%).

### Direct pairwise comparison

#### Major adverse cardiovascular events

**Fig. 3:**
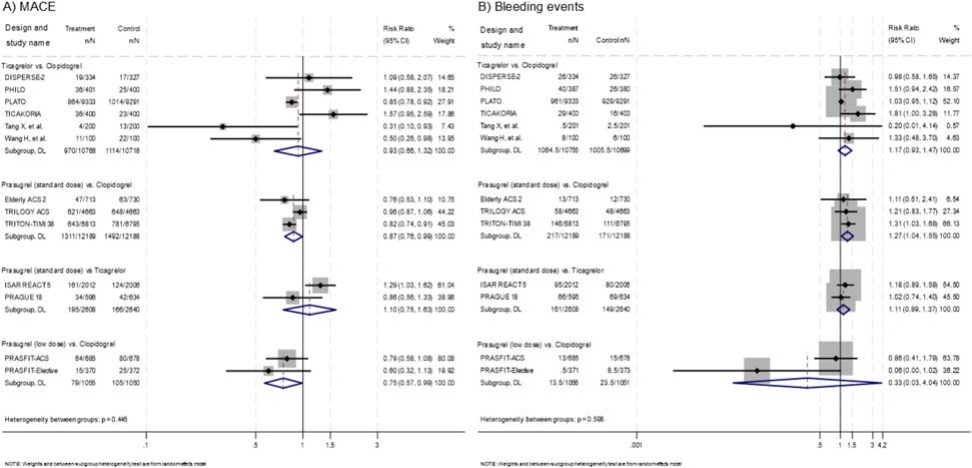
Direct pairwise comparisons of randomized trials for major adverse cardiovascular events (MACE) and bleeding events. The size of the square box is proportional to the weight of each trial in the meta-analysis. The overall estimate and confidence interval (CI) are marked by a diamond

Figure 3A shows the results of a direct pairwise meta-analysis for MACE. Six trials compared the MACE between ticagrelor and clopidogrel, showing no significant difference in pooled effect sizes (RR 0.93, 95% CI 0.66–1.32; *I*^*2*^ = 68.9%; Cochran’s *Q* = 16.08, *P* < 0.01) [7, 16, 22–25]. Among these trials, three comparing prasugrel (standard dose) to clopidogrel showed the superiority of prasugrel (standard dose) (RR 0.87, 95% CI 0.76–0.99; *I*^*2*^ = 61.1%; Cochran’s *Q* = 5.14, *P* = 0.08) [19–21]. Two trials comparing prasugrel (standard dose) to ticagrelor showed equivalent efficacy (RR 1.10, 95% CI 0.75–1.63; *I*^*2*^ = 61.9%; Cochran’s *Q* = 2.63, *P* = 0.11) [26, 27]. Finally, two trials comparing prasugrel (low dose) to clopidogrel indicated the superiority of prasugrel (low dose) (RR 0.75, 95% CI 0.57–0.99; *I*^*2*^ = 0%; Cochran’s *Q* = 0.59, *P* = 0.44) [17, 18].

#### Bleeding events

Figure 3B shows the results of a direct pairwise meta-analysis for bleeding events. Six trials compared bleeding events between ticagrelor and clopidogrel, showing no significant difference (RR 1.17, 95% CI 0.93–1.47; *I*^*2*^ = 29.9%; Cochran’s *Q* = 7.14, *P* = 0.21) [7, 16, 22–25]. Three trials comparing prasugrel (standard dose) to clopidogrel demonstrated the inferiority of prasugrel (standard dose) (RR 1.27, 95% CI 1.04–1.55; *I*^*2*^ = 0%; Cochran’s *Q* = 0.25, *P* = 0.88) [19–21]. Two trials comparing prasugrel (standard dose) to ticagrelor showed equivalent safety (RR 1.11, 95% CI 0.89–1.37; *I*^*2*^ = 0%; Cochran’s *Q* = 0.47, *P* = 0.49) [26, 27]. Two trials comparing prasugrel (low dose) to clopidogrel showed no significant difference (RR 0.33, 95% CI 0.03–4.04; *I*^*2*^ = 68.5%; Cochran’s *Q* = 3.17, *P* = 0.08) [17, 18].

#### Cardiovascular death

Supplemental Figure 2a shows the results of a direct pairwise meta-analysis for cardiovascular death. Six trials compared the bleeding events between ticagrelor and clopidogrel, showing no significant difference (RR 0.97, 95% CI 0.63–1.51; *I*^*2*^ = 45.4%; Cochran’s *Q* = 9.15, *P* = 0.10) [7, 16, 22–25]. Three trials comparing prasugrel (standard dose) to clopidogrel also showed no significant difference (RR 0.92, 95% CI 0.81–1.03; *I*^*2*^ = 0%; Cochran’s *Q* = 0.21, *P* = 0.90) [19–21]. Two trials comparing prasugrel (standard dose) to ticagrelor had equivalent outcomes (RR 1.03, 95% CI 0.76–1.39; *I*^*2*^ = 0%; Cochran’s *Q* = 0.18, *P* = 0.67) [26, 27]. Two trials comparing prasugrel (low dose) to clopidogrel showed no significant difference (RR 1.45, 95% CI 0.54–3.91; *I*^*2*^ = 0%; Cochran’s *Q* = 0.04, *P* = 0.85) [17, 18].

#### Myocardial infarction

Supplemental Figure 2b shows the results of a direct pairwise meta-analysis for MI. Six trials compared the bleeding events between ticagrelor and clopidogrel, showing no significant difference (RR 0.90, 95% CI 0.64–1.27; *I*^*2*^ = 46.2%; Cochran’s *Q* = 9.30, *P* = 0.10) [7, 16, 22–25]. Three trials comparing prasugrel (standard dose) to clopidogrel also showed no significant difference (RR 0.85, 95% CI 0.70–1.03; *I*^*2*^ = 68.1%; Cochran’s *Q* = 6.28, *P* = 0.04) [19–21]. Two trials comparing prasugrel (standard dose) to ticagrelor had equivalent outcomes (RR 1.24, 95% CI 0.67–2.29; *I*^*2*^ = 65.5%; Cochran’s *Q* = 2.90, *P* = 0.09). [26, 27] Two trials comparing prasugrel (low dose) to clopidogrel showed no significant difference (RR 0.79, 95% CI 0.58–1.09; *I*^*2*^ = 0%; Cochran’s *Q* = 0.42, *P* = 0.52). [17, 18].

#### Stroke

Supplemental Figure 2c shows the results of a direct pairwise meta-analysis for stroke. Six trials compared the bleeding events between ticagrelor and clopidogrel, showing no significant difference (RR 1.16; 95% CI 0.91–1.47; *I*^*2*^ = 0%; Cochran’s *Q* = 3.41, *P* = 0.64) [7, 16, 22–25]. Three trials comparing prasugrel (standard dose) to clopidogrel also showed no significant difference (RR 0.92, 95% CI 0.72–1.16; *I*^*2*^ = 0.0%; Cochran’s *Q* = 1.52, *P* = 0.47) [19–21]. Two trials comparing prasugrel (standard dose) to ticagrelor had equivalent outcomes (RR 1.02, 95% CI, 0.59–1.75; *I*^*2*^ = 0.0%; Cochran’s *Q* = 0.85, *P* = 0.36) [26, 27]. Two trials comparing prasugrel (low dose) to clopidogrel showed no significant difference (RR 0.70, 95% CI 0.21–2.34; *I*^*2*^ = 19.1%; Cochran’s *Q* = 1.24, *P* = 0.27) [17, 18].

### Network meta-analysis

#### Contribution of each direct comparison

The percentage contributions of each direct comparison in the entire network to the overall estimates of indirect comparisons were visually illustrated in a contribution plot (Supplemental Figure 3). The percentage contributions were 31.6% for clopidogrel vs. prasugrel (low dose), 34.2% for clopidogrel vs. prasugrel (standard dose), 19.0% for clopidogrel vs. ticagrelor, and 15.2% for prasugrel (standard dose) vs. ticagrelor, respectively.

#### Major adverse cardiovascular events

**Fig. 4:**
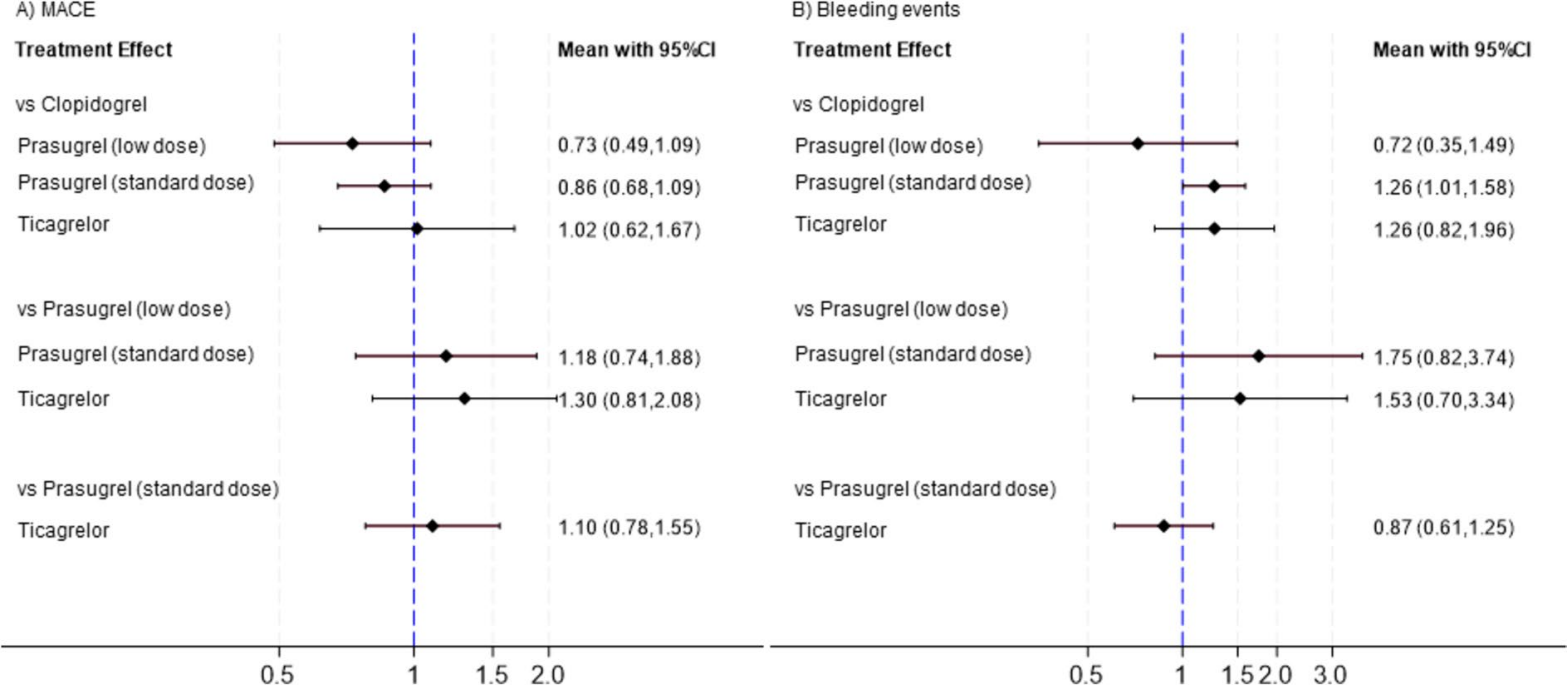
Network meta-analysis (NMA) for MACE and bleeding events. This forest plot illustrates the estimated risk ratio and 95% CIs for all comparisons of antiplatelet agents obtained through NMA

Figure 4A shows the results of NWA for the primary efficacy endpoint of MACE. Compared with clopidogrel, prasugrel (low dose), prasugrel (standard dose), and ticagrelor showed no significant difference (RR, 0.73; 95% CI 0.49–1.09; RR, 0.86; 95% CI 0.68–1.09; RR, 1.02; 95% CI 0.62–1.67, respectively). Comparisons between prasugrel (low dose), prasugrel (standard dose), and ticagrelor also showed no significant difference (RR, 1.18; 95% CI 0.74–1.88; RR, 1.30; 95% CI 0.81–2.08, respectively). When prasugrel (standard dose) was set as the reference, ticagrelor also showed no significant difference (RR, 1.10; 95% CI 0.78–1.55) (Supplemental Figure 4). RRs for differences across antiplatelet agents are summarized in a league table (Supplemental Table 5a).

#### Bleeding events

Figure 4B shows the results of NWA for the secondary safety endpoint of bleeding. Compared to clopidogrel, prasugrel (standard dose) showed significant inferiority (RR, 1.26; 95% CI 1.01–1.58), whereas prasugrel (low dose) and ticagrelor showed no significant difference (RR, 0.72; 95% CI 0.35–1.49; RR, 1.26; 95% CI 0.82–1.96, respectively). Comparisons between prasugrel (low dose), prasugrel (standard dose), and ticagrelor also showed no significant difference (RR, 1.75; 95% CI 0.82–3.74; RR, 1.53; 95% CI 0.70–3.34, respectively). When prasugrel (standard dose) was used as the reference, ticagrelor showed no significant difference (RR, 0.87; 95% CI 0.61–1.25) (Supplemental Figure 5). RRs for the differences across antiplatelet agents are summarized in a league table (Supplemental Table 5b).

#### Cardiovascular death

Supplemental Figure 6 and 7 show the results of NWA for cardiovascular death. Compared to clopidogrel, prasugrel (low dose), prasugrel (standard dose), and ticagrelor showed no significant difference (RR, 1.45; 95% CI 0.54–3.91; RR, 0.92; 95% CI 0.81–1.03; RR, 1.06; 95% CI 0.76–1.48, respectively). Comparisons between prasugrel (low dose), prasugrel (standard dose), and ticagrelor also showed no significant difference (RR, 0.63; 95% CI 0.23–1.72; RR, 0.61; 95% CI 0.23–1.66, respectively). When prasugrel (standard dose) was used as the reference, ticagrelor showed no significant difference (RR, 0.97; 95% CI 0.84–1.11). RRs for the differences across antiplatelet agents are summarized in a league table (Supplemental Table 5c).

#### Myocardial infarction

Supplemental Figure 8 and 9 show the results of NWA for MI. Compared to clopidogrel, prasugrel (low dose), prasugrel (standard dose), and ticagrelor showed no significant difference (RR, 0.81; 95% CI 0.54–1.22; RR, 0.84; 95% CI 0.67–1.06; RR, 1.27; 95% CI 0.74–2.16, respectively). Comparisons between prasugrel (low dose), prasugrel (standard dose), and ticagrelor also showed no significant difference (RR, 1.04; 95% CI 0.65–1.67; RR, 1.11; 95% CI 0.69–1.79, respectively). When prasugrel (standard dose) was used as the reference, ticagrelor showed no significant difference (RR, 1.06; 95% CI 0.75–1.49). RRs for the differences across antiplatelet agents are summarized in a league table (Supplemental Table 5 d).

#### Stroke

Supplemental Figure 10 and 11 show the results of NWA for stroke. Compared to clopidogrel, prasugrel (low dose), prasugrel (standard dose), and ticagrelor showed no significant difference (RR, 0.67; 95% CI 0.23–1.97; RR, 0.92; 95% CI 0.72–1.16; RR, 0.80; 95% CI 0.42–1.53, respectively). Comparisons between prasugrel (low dose), prasugrel (standard dose), and ticagrelor also showed no significant difference (RR, 1.36; 95% CI 0.45–4.10; RR, 1.72; 95% CI 0.57–5.18, respectively). When prasugrel (standard dose) was used as the reference, ticagrelor showed no significant difference (RR, 1.26; 95% CI 0.90–1.77). RRs for the differences across the antiplatelet agents are summarized in a league table (Supplemental Table 5e).

### Ranking of antiplatelet agents

**Fig. 5:**
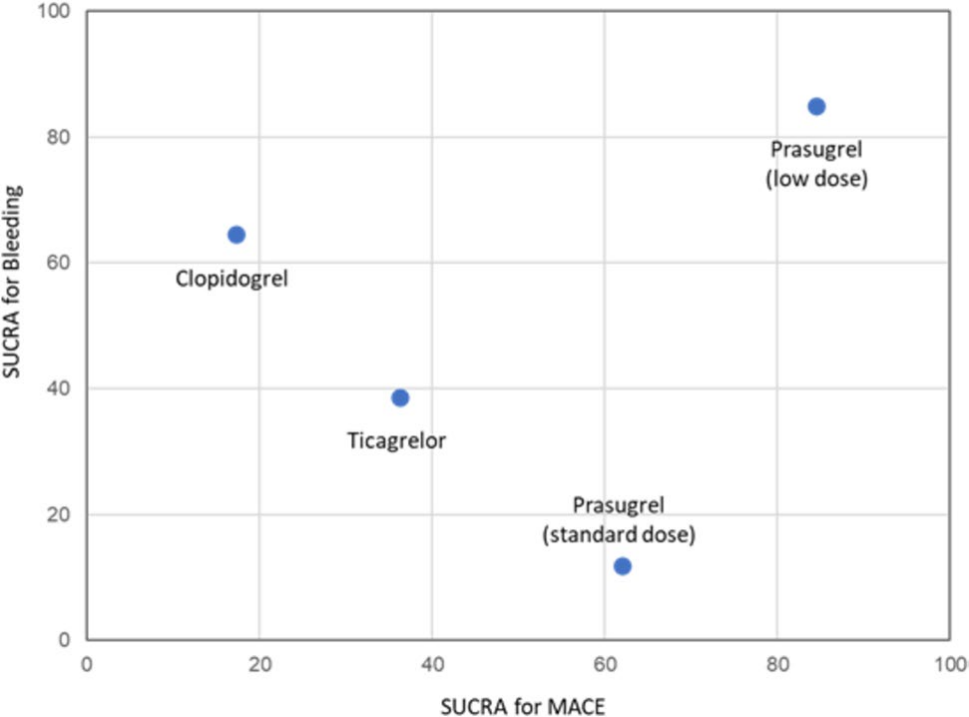
Cluster rank plot of MACE and bleeding events. This cluster rank plot shows antiplatelet agents ranked based on the surface under the cumulative ranking curve (SUCRA) values for MACE and bleeding events. Agents on the right side of the *x*-axis have higher efficacy (lower MACE), while those on the top of the *y*-axis have higher safety (lower bleeding)

The probability ranking of MACE and bleeding events among antiplatelet agents was assessed. Table 2 displays the SUCRA values, indicating the probability of each treatment being the best, and the mean rank, which represents the average ranking probability. Figure 5 shows the results of SUCRA clustering for treatments in the network based on MACE and bleeding events, with interventions closer to the upper right considered more favorable. Low-dose prasugrel demonstrated the most favorable profile for both MACE and bleeding events.

**Table 2:** Results of surface under the cumulative ranking curves

	MACE			Bleeding		
	SUCRA	P best	Mean rank	SUCRA	P best	Mean rank
Clopidogrel	17.3	0.5	3.5	64.6	15.3	2.1
Prasugrel (low dose)	84.5	70.3	1.5	84.9	77.7	1.5
Prasugrel (standard dose)	62.0	21.4	2.1	11.8	0.5	3.6
Ticagrelor	36.2	7.8	2.9	38.7	6.5	2.8

*SUCRA* surface under the cumulative ranking curve, *P best* probability of each treatment being the best

### Sensitivity analyses

Two sensitivity analyses were conducted, excluding two small-sample trials: Tang et al. and Wang et al [24, 25]. Furthermore, three trials with high RoB were excluded: Elderly ACS 2, Tang et al., and Wang et al [19, 24, 25]. These sensitivity analyses were consistent with the primary analysis, suggesting the robustness of our findings to potential biases and variations in study characteristics (Supplemental Figure 12a–e and 13a–e).

### Inconsistency and publication bias

The global test for inconsistency yielded a P value of 0.95, indicating no evidence of inconsistency. When comparing each node, the difference between direct and indirect comparison results was not statistically significant, as shown in Supplemental Table 6, indicating no evidence of local inconsistency. Therefore, the consistency assumption was accepted, because inconsistency was absent in both global and local tests.

The funnel plot for both MACE and bleeding events shows an overall symmetrical distribution of studies on either side of the middle line in the upper section, suggesting a reduced probability of publication bias in prominent publications. Although a scattered point was observed at the lower end of the funnel plot, it did not imply the presence of a small sample effect (Egger’s test, *P* = 0.45 for MACE, Supplemental Figure 14a; *P* = 0.97 for bleeding events, Supplemental Figure 14b).

## Discussion

This NMA, comprising 13 RCTs, investigated the efficacy and safety of low-dose prasugrel with other P2Y12 receptor inhibitors when used in dual antiplatelet therapy with aspirin. The standard dose of prasugrel was associated with a significantly higher risk of bleeding events compared to clopidogrel, whereas no significant differences were found in overall MACE, cardiovascular death, MI, or stroke across the P2Y12 receptor inhibitors. The ranking probabilities from the SUCRA analysis suggested a potential advantage for low-dose prasugrel in both reducing MACE and bleeding events.

The first RCT comparing prasugrel with clopidogrel, TRITON-TIMI 38, demonstrated that prasugrel reduced rates of ischemic events, including stent thrombosis, but was associated with an increased risk of major bleeding, including fatal bleeding [21]. Subsequent RCTs, including PRAGUE 18, TRILOGY-ACS, Elderly ACS 2, and ISAR REACT 5, explored the use of an adjusted 5-mg dose of prasugrel based on age or body weight, in addition to the 10-mg dose [19, 20, 26, 27]. Notably, TRILOGY-ACS and Elderly ACS 2 showed similar rates of MACE and bleeding events for both prasugrel and clopidogrel [19, 20]. These findings contrast with those of TRITON-TIMI 38, suggesting that dose adjustment of prasugrel could significantly influence the balance between efficacy and safety [21]. In the ISAR REACT 5 trial, which used an adjusted 5 mg dose of prasugrel, ticagrelor, considered the most potent P2Y12 receptor inhibitor, demonstrated equivalent bleeding events [hazard ratio (HR), 1.12; 95% CI 0.83–1.51; *P* = 0.46] compared to prasugrel [26]. However, ticagrelor exhibited inferior MACE rates (HR, 1.36; 95% CI 1.09–1.70; *P* = 0.006) compared to prasugrel. Given that ticagrelor has the most potent platelet inhibition among P2Y12 receptor inhibitors, this suggests potential for further optimizing the balance between MACE and bleeding with an adjusted prasugrel dose.

Previous NMAs comparing the efficacy and safety of P2Y12 receptor inhibitors in patients with non ST-elevation MI confirmed that prasugrel was associated with a significantly lower risk of MACE compared to clopidogrel (HR, 0.84; 95% CI 0.71–0.99). In contrast, ticagrelor did not show a statistically significant benefit in terms of MACE reduction compared to clopidogrel (HR, 1.02; 95% CI 0.84–1.23). Regarding bleeding events, prasugrel showed no significant difference in risk compared to clopidogrel (HR, 1.30; 95% CI 0.97–1.74). However, ticagrelor was associated with a significantly higher risk of bleeding events compared to clopidogrel (HR, 1.33; 95% CI 1.00–1.77). Notably, there were no statistically significant differences in either MACE (HR, 0.83; 95% CI 0.66–1.04) or bleeding risk (HR, 0.97; 95% CI 0.69–1.38) between prasugrel and ticagrelor [28].

Previous meta-analyses or RCTs have evaluated the efficacy and safety of P2Y12 receptor inhibitors, indicating that prasugrel and ticagrelor may be more effective than clopidogrel but with potentially less favorable safety profiles. In addition, no significant difference in efficacy or safety was observed between prasugrel and ticagrelor. A key limitation of these prior analyses was the aggregation of data from studies using different prasugrel doses under a single category of “prasugrel”. Our present analysis addresses this limitation by distinguishing between standard-dose and low-dose prasugrel. Therefore, this analysis may provide novel insights into P2Y12 receptor inhibitor research by exploring the potential impact of prasugrel dosage on clinical outcomes.

A fixed dose of 10-mg prasugrel may be excessive for some patients in terms of antiplatelet inhibition. An adjusted dose of 5 mg appears reasonable when prioritizing effectiveness, whereas a 3.75-mg dose might be safer. It is important to consider the potential for variability in response to agents across studies due to differences in baseline characteristics of study populations, such as race, age, or body frame. Low-dose prasugrel may be a promising option with more balanced platelet inhibition compared to standard-dose prasugrel or other P2Y12 receptor inhibitors. PRASFIT-ACS and PRASFIT-Elective investigated the use of 3.75 mg prasugrel compared to clopidogrel in patients with ACS and elective percutaneous coronary intervention, respectively [17, 18]. In PRASFIT-ACS, MACE occurred in 9.4% with prasugrel and 11.8% with clopidogrel (HR, 0.77; 95% CI 0.56–1.07), and bleeding events occurred in 1.9% with prasugrel and 2.2% with clopidogrel (HR, 0.82; 95% CI 0.39–1.73). [17] Similarly, PRASFIT-Elective showed trends toward lower MACE (4.1% vs. 6.7%) and bleeding events (0% vs. 2.2%) with prasugrel [18]. Both trials showed trends toward lower rates of MACE and bleeding events with low-dose prasugrel, but these differences were not statistically significant. These trials investigating low-dose prasugrel demonstrated its potential to mitigate excessive antiplatelet inhibition observed with standard-dose prasugrel in the TRITON-TIMI 38 trial, suggesting a balance between efficacy and safety [21].

In our NWA, prasugrel did not show a statistically significant difference in MACE compared to other agents, regardless of dose. Direct comparisons suggested that the standard dose of prasugrel was associated with a significantly higher risk of bleeding events compared to clopidogrel, and this finding was also confirmed in the NMA. In direct comparison with clopidogrel, standard-dose prasugrel showed a statistically significant difference in terms of both MACE and bleeding events. Ticagrelor did not show a statistically significant difference in either outcome, whether through direct comparison or NMA. Furthermore, there were no significant differences between standard-dose prasugrel and ticagrelor for either MACE or bleeding events. The variability in ticagrelor’s effects is likely attributable to the wide variation in results from RCTs. The PLATO trial demonstrated a significantly lower incidence of MACE with ticagrelor compared to clopidogrel (9.8% vs. 11.7%; *P* < 0.001) [7]. However, results from PHILO and TICAKOREA, which had similar study designs to PLATO in terms of comparing ticagrelor and clopidogrel, showed opposite trends with no significant differences [22, 23]. Notably, unlike PLATO where over 90% of participants were white, PHILO and TICAKOREA primarily included East Asian patients, suggesting a potential influence of ethnicity on treatment response. The superior efficacy of standard-dose prasugrel in terms of MACE observed in direct comparisons with clopidogrel appeared to extend to the low-dose group as well. However, in the NMA, this potential benefit shown in direct comparisons was obscured by indirect estimation. In addition, the safety profile of low-dose prasugrel was not definitively established in either direct comparisons or the NMA. Notably, the PRASFIT-Elective trial had a relatively small sample size for the number of bleeding events [18]. Although the prasugrel arm reported no bleeding events (0% vs. 2.2%), this resulted in a wide CI in our forest plot, limiting the generalizability of the findings. Further large-scale RCTs investigating low-dose prasugrel are warranted to verify its potential advantages in terms of efficacy and safety.

When interpreting this NMA, it is important to acknowledge that ACS and chronic coronary syndrome were analyzed together without distinction. These two conditions exhibit different responses to antiplatelet therapy, and particularly in the acute phase of ACS, both ischemic and bleeding risks are elevated and dynamically fluctuate over time [29]. While this NMA provides valuable insights into the positioning of low-dose prasugrel, its generalizability is limited, given the unique characteristics of ACS. Therefore, further RCTs on low-dose prasugrel are warranted. Furthermore, the HOST-REDUCE-POLYTECH-ACS trial conducted in Korea demonstrated that reducing the prasugrel dose from 10 to 5 mg 1 month after PCI improved safety without compromising efficacy [30]. This finding suggests that low-dose prasugrel may be a more favorable option in East Asian populations. On the other hand, while this NMA indicates that low-dose prasugrel may achieve a favorable balance between ischemic and bleeding risks, it is crucial to recognize that the supporting data were derived exclusively from RCTs conducted in Japan. Ethnic differences in the pharmacodynamics and clinical outcomes of P2Y12 inhibitors have been reported, with East Asian patients exhibiting greater responsiveness to P2Y12 inhibitors than White populations, a phenomenon known as the “East-Asian paradox.” Given this, low-dose prasugrel may increase the risk of ischemic events in White patients. Thus, the generalizability of this NMA’s findings to other ethnic groups is limited, highlighting the need for further investigations in non-Japanese populations.

This study had several limitations that need to be considered when interpreting its findings. The NMA was conducted solely based on existing study-level data and not on individual patient data, possibly affecting the design of the included trials. Second, although this NMA included 13 RCTs, it may have been limited in providing robust conclusions. In each study, registrations were from various countries, and because the response to antiplatelet drugs differs among ethnicities, variability in results between studies may have occurred. To partially overcome the limitation of heterogeneity among studies for the primary and secondary outcomes of MACE and all bleeding, the more conservative random-effects model was used for these outcomes. In particular, there were only two RCTs concerning low-dose prasugrel, both of which were conducted in Japan. Although large-sample trials such as PLATO and TRITON-TIMI 38 were included, the use of a random-effects model appropriately accounted for interstudy heterogeneity, incorporated study-level variability, minimized potential bias, and ensured the robustness of the results.

In conclusion, no significant differences were observed in MACE or bleeding events among the four P2Y12 receptor inhibitors (clopidogrel, low-dose prasugrel, standard-dose prasugrel, and ticagrelor) evaluated as DAPT in combination with aspirin. However, the ranking probabilities suggested that low-dose prasugrel may have the most favorable efficacy and safety profile. Further RCTs investigating low doses of prasugrel are needed to obtain more robust results.

## Supplementary Information

Below is the link to the electronic supplementary material.

Supplementary file1 (DOCX 1287 KB)
