# Supplementary material for: Efficacy and safety of low-dose prasugrel as dual antiplatelet therapy in patients with ischemic heart disease: a systematic review and network meta-analysis of randomized controlled trials
Source: Cardiovasc Interv Ther. 2025 Jun 9;40(4):767–77. doi: 10.1007/s12928-025-01129-2 (PMC12431874; doi:10.1007/s12928-025-01129-2)

Supplemental Materials

Efficacy and Safety of Low-Dose Prasugrel as Dual Antiplatelet Therapy in Patients with Ischemic Heart Disease: A Systematic Review and Network Meta-Analysis of Randomized Controlled Trials

Toshiharu Fujii et al.

**Table of Contents**

Supplemental Table 1. PRISMA-NMA checklist

Supplemental Table 2a. PubMed search strategy

Supplemental Table 2b. Cochrane Trials search strategy

Supplemental Table 2c. Scopus search strategy

Supplemental Table 3a. Definitions of trial-defined MACE

Supplemental Table 3b. Definitions of trial-defined bleeding events

Supplemental Table 3c. Definitions of trial-defined death

Supplemental Table 3d. Definitions of trial-defined MI

Supplemental Table 3e. Definitions of trial-defined stroke

Supplemental Table 4a. Criteria for inclusion and exclusion in individual trials

Supplemental Table 4b. Baseline characteristics of included trials

Supplemental Table 4c. Therapeutic interventions in individual trials

Supplemental Table 5a. League table for MACE

Supplemental Table 5b. League table for bleeding events

Supplemental Table 5c. League table for CV death

Supplemental Table 5d. League table for MI

Supplemental Table 5e. League table for stroke

Supplemental Table 6. Local inconsistency test between direct and indirect treatment comparisons in mixed treatment comparison

Supplemental Figure 1a. Cochrane risk of bias assessment

Supplemental Figure 1b. Cochrane risk of bias graph presented as percentages

Supplemental Figure 2a. Direct pairwise meta-analysis of randomized trials for CV death

Supplemental Figure 2b. Direct pairwise meta-analysis of randomized trials for MI

Supplemental Figure 2c. Direct pairwise meta-analysis of randomized trials for stroke

Supplemental Figure 3. Contribution plot

Supplemental Figure 4. Forest plot of network meta-analysis for MACE

Supplemental Figure 5. Forest plot of network meta-analysis for bleeding events

Supplemental Figure 6. Network meta-analysis for CV death

Supplemental Figure 7. Forest plot of network meta-analysis for CV death

Supplemental Figure 8. Network meta-analysis for MI

Supplemental Figure 9. Forest plot of network meta-analysis for MI

Supplemental Figure 10. Network meta-analysis for stroke

Supplemental Figure 11. Forest plot of network meta-analysis for stroke

Supplemental Figure 12a. Sensitivity analysis: Excluding trials with a high risk of bias, MACE

Supplemental Figure 12b. Sensitivity analysis: Excluding trials with a high risk of bias, bleeding events

Supplemental Figure 12c. Sensitivity analysis: Excluding trials with a high risk of bias, CV death

Supplemental Figure 12d. Sensitivity analysis: Excluding trials with a high risk of bias, MI

Supplemental Figure 12e. Sensitivity analysis: Excluding trials with a high risk of bias, stroke

Supplemental Figure 13a. Sensitivity analysis: Excluding small-sample trials, MACE

Supplemental Figure 13b. Sensitivity analysis: Excluding small-sample trials, bleeding events

Supplemental Figure 13c. Sensitivity analysis: Excluding small-sample trials, CV death

Supplemental Figure 13d. Sensitivity analysis: Excluding small-sample trials, MI

Supplemental Figure 13e. Sensitivity analysis: Excluding small-sample trials, stroke

Supplemental Figure 14a. Funnel plot for MACE

Supplemental Figure 14b. Funnel plot for bleeding events

Supplemental Table 1. PRISMA-NMA checklist

| **Section/Topic** | **Item #** | **Checklist Item** | **Reported on Page #** |
| --- | --- | --- | --- |
| **TITLE** |  |  |  |
| Title | 1 | Identify the report as a systematic review *incorporating a network meta-analysis (or related form of meta-analysis).* | #1 |
|  |  |  |  |
| **ABSTRACT** |  |  |  |
| Structured summary | 2 | Provide a structured summary including, as applicable:  **Background:** main objectives  **Methods:** data sources; study eligibility criteria, participants, and interventions; study appraisal; and *synthesis methods, such as network meta-analysis.*  **Results:** number of studies and participants identified; summary estimates with corresponding confidence/credible intervals; *treatment rankings may also be discussed. Authors may choose to summarize pairwise comparisons against a chosen treatment included in their analyses for brevity.*  **Discussion/Conclusions:** limitations; conclusions and implications of findings.  **Other:** primary source of funding; systematic review registration number with registry name. | #2-3 |
|  |  |  |  |
| **INTRODUCTION** |  |  |  |
| Rationale | 3 | Describe the rationale for the review in the context of what is already known*, including mention of why a network meta-analysis has been conducted.* | #5-6 |
| Objectives | 4 | Provide an explicit statement of questions being addressed, with reference to participants, interventions, comparisons, outcomes, and study design (PICOS). | #5-6 |
|  |  |  |  |
| **METHODS** |  |  |  |
| Protocol and registration | 5 | Indicate whether a review protocol exists and if and where it can be accessed (e.g., Web address); and, if available, provide registration information, including registration number. | #6–7 |
| Eligibility criteria | 6 | Specify study characteristics (e.g., PICOS, length of follow-up) and report characteristics (e.g., years considered, language, publication status) used as criteria for eligibility, giving rationale. *Clearly describe eligible treatments included in the treatment network, and note whether any have been clustered or merged into the same node (with justification).* | #6-8, S Table 2a–c, Figure 1 |
| Information sources | 7 | Describe all information sources (e.g., databases with dates of coverage, contact with study authors to identify additional studies) in the search and date last searched. | #6-8, Table S 2a–c |
| Search | 8 | Present full electronic search strategy for at least one database, including any limits used, such that it could be repeated. | #S Table 2a–c |
| Study selection | 9 | State the process for selecting studies (i.e., screening, eligibility, included in systematic review, and, if applicable, included in the meta-analysis). | #6-8, S Table 2a–c, Figure 1 |
| Data collection process | 10 | Describe method of data extraction from reports (e.g., piloted forms, independently, in duplicate) and any processes for obtaining and confirming data from investigators. | #6-8, S Table 2a–c |
| Data items | 11 | List and define all variables for which data were sought (e.g., PICOS, funding sources) and any assumptions and simplifications made. | #8-9, S Table 3a-e and 4a |
| **Geometry of the network** | **S1** | Describe methods used to explore the geometry of the treatment network under study and potential biases related to it. This should include how the evidence base has been graphically summarized for presentation, and what characteristics were compiled and used to describe the evidence base to readers. | #9-10 |
| Risk of bias within individual studies | 12 | Describe methods used for assessing risk of bias of individual studies (including specification of whether this was done at the study or outcome level), and how this information is to be used in any data synthesis. | #8 |
| Summary measures | 13 | State the principal summary measures (e.g., risk ratio, difference in means). *Also describe the use of additional summary measures assessed, such as treatment rankings and surface under the cumulative ranking curve (SUCRA) values, as well as modified approaches used to present summary findings from meta-analyses.* | #9-10 |
| Planned methods of analysis | 14 | Describe the methods of handling data and combining results of studies for each network meta-analysis. This should include, but not be limited to:   - *Handling of multi-arm trials;* - *Selection of variance structure;* - *Selection of prior distributions in Bayesian analyses; and* - *Assessment of model fit.* | #9-10 |
| **Assessment of Inconsistency** | **S2** | Describe the statistical methods used to evaluate the agreement of direct and indirect evidence in the treatment network(s) studied. Describe efforts taken to address its presence when found. | #9-10 |
| Risk of bias across studies | 15 | Specify any assessment of risk of bias that may affect the cumulative evidence (e.g., publication bias, selective reporting within studies). | #9-10 |
| Additional analyses | 16 | Describe methods of additional analyses if done, indicating which were pre-specified. This may include, but not be limited to, the following:   - Sensitivity or subgroup analyses; - Meta-regression analyses; - *Alternative formulations of the treatment network; and* - *Use of alternative prior distributions for Bayesian analyses (if applicable).* | #9-10 |
|  |  |  |  |
| **RESULTS** |  |  |  |
| Study selection | 17 | Give numbers of studies screened, assessed for eligibility, and included in the review, with reasons for exclusions at each stage, ideally with a flow diagram. | #10-11, Figure 1 |
| **Presentation of network structure** | **S3** | Provide a network graph of the included studies to enable visualization of the geometry of the treatment network. | Figure 2 |
| **Summary of network geometry** | **S4** | Provide a brief overview of characteristics of the treatment network. This may include commentary on the abundance of trials and randomized patients for the different interventions and pairwise comparisons in the network, gaps of evidence in the treatment network, and potential biases reflected by the network structure. | #10-11, Figure 3 and 4, and S Table 4b-c |
| Study characteristics | 18 | For each study, present characteristics for which data were extracted (e.g., study size, PICOS, follow-up period) and provide the citations. | Table 1 and S Table 4b-c |
| Risk of bias within studies | 19 | Present data on risk of bias of each study and, if available, any outcome level assessment. | #11-12 and S Figure 1a–b |
| Results of individual studies | 20 | For all outcomes considered (benefits or harms), present, for each study: 1) simple summary data for each intervention group, and 2) effect estimates and confidence intervals. *Modified approaches may be needed to deal with information from larger networks.* | #10-12 and S Table 4b–c |
| Synthesis of results | 21 | Present results of each meta-analysis done, including confidence/credible intervals. *In larger networks, authors may focus on comparisons versus a particular comparator (e.g. placebo or standard care), with full findings presented in an appendix. League tables and forest plots may be considered to summarize pairwise comparisons.* If additional summary measures were explored (such as treatment rankings), these should also be presented. | #12–18, Figure 3-4, S Table 5a–e, and S Figure 2–11 |
| **Exploration for inconsistency** | **S5** | Describe results from investigations of inconsistency. This may include such information as measures of model fit to compare consistency and inconsistency models, *P* values from statistical tests, or summary of inconsistency estimates from different parts of the treatment network. | #20, S Table 6 |
| Risk of bias across studies | 22 | Present results of any assessment of risk of bias across studies for the evidence base being studied. | #11-12 and S Figure 1a–b |
| Results of additional analyses | 23 | Give results of additional analyses, if done (e.g., sensitivity or subgroup analyses, meta-regression analyses*, alternative network geometries studied, alternative choice of prior distributions for Bayesian analyses,* and so forth). | #19 and S Figure 12-13 |
|  |  |  |  |
| **DISCUSSION** |  |  |  |
| Summary of evidence | 24 | Summarize the main findings, including the strength of evidence for each main outcome; consider their relevance to key groups (e.g., healthcare providers, users, and policy-makers). | #20-21 |
| Limitations | 25 | Discuss limitations at study and outcome level (e.g., risk of bias), and at review level (e.g., incomplete retrieval of identified research, reporting bias). *Comment on the validity of the assumptions, such as transitivity and consistency. Comment on any concerns regarding network geometry (e.g., avoidance of certain comparisons).* | #25-26 |
| Conclusions | 26 | Provide a general interpretation of the results in the context of other evidence, and implications for future research. | #26 |
|  |  |  |  |
| **FUNDING** |  |  |  |
| Funding | 27 | Describe sources of funding for the systematic review and other support (e.g., supply of data); role of funders for the systematic review. This should also include information regarding whether funding has been received from manufacturers of treatments in the network and/or whether some of the authors are content experts with professional conflicts of interest that could affect use of treatments in the network. | #29 |

PICOS = population, intervention, comparators, outcomes, and study design

Supplemental Table 2a. PubMed search strategy

Data collection period: 1998 to November 1, 2023

Search date: November 1, 2023

No language restrictions

|  | Query |
| --- | --- |
| #1 | “Myocardial ischemia”[mh] |
| #2 | (“ischemic heart disease”[tiab]) OR (“chronic coronary syndrome”[tiab]) |
| #3 | (“angina pectoris”[tiab]) OR (“stable angina pectoris” [tiab]) OR (“stable angina”[tiab]) |
| #4 | (“unstable angina”[tiab]) OR (“unstable angina pectoris”[tiab]) |
| #5 | (“acute coronary syndrome”[tiab]) OR (“myocardial infarction”[tiab]) OR (“acute myocardial infarction”[tiab]) |
| #6 | (“ST-elevation Myocardial Infarction”[tiab]) OR (“ST-segment elevation myocardial infarction”[tiab]) |
| #7 | (“Non-ST-elevation myocardial infarction”[tiab]) OR (“Non-ST-segment elevation myocardial infarction”[tiab]) |
| #8 | #1 OR #2 OR #3 OR #4 OR #5 OR #6 OR #7 |
| #9 | (“Dual Antiplatelet Therapy”[mh]) OR (“dual antiplatelet therapy”[tiab]) |
| #10 | (“Clopidogrel”[mh]) OR (“clopidogrel”[tiab]) |
| #11 | (“Prasugrel Hydrochloride”[mh]) OR (“prasugrel”[tiab]) |
| #12 | (“Ticagrelor”[mh]) OR (“ticagrelor”[tiab]) |
| #13 | (“Aspirin”[mh]) OR (“aspirin”[tiab]) |
| #14 | #9 AND (#10 OR #11 OR #12 OR #13) |
| #15 | (“Death”[mh]) OR (“death”[tiab]) OR (“mortality”[tiab]) |
| #16 | (“Cerebrovascular Disorders”[mh]) OR (“stroke”[tiab]) OR (“cerebral infarction”[tiab]) |
| #17 | (“Coronary Thrombosis”[mh]) OR (“coronary thrombosis”[tiab]) OR (“stent thrombosis”[tiab]) |
| #18 | (“Hemorrhage”[mh]) OR (“hemorrhage”[tiab]) OR (“bleeding”[tiab]) |
| #19 | #15 OR #16 OR #17 OR #18 |
| #20 | (“Randomized Controlled Trial”[pt]) OR (“randomized controlled trial”[tiab]) OR (“randomized trial”[tiab]) OR (“randomized”[tiab]) |
| #21 | “1998/01/01”[DP] : “3000”[DP] |
| #22 | (Anticoagulants[mh]) OR (edoxaban[tiab]) OR (rivaroxaban[tiab]) OR (apixaban[tiab]) OR (dabigatran[tiab]) OR (warfarin[tiab]) OR (“cilostazol”[tiab]) |
| #23 | (“Vascular Grafting”[mh]) OR (bypass[ti]) OR (graft[ti]) |
| #24 | (Review[pt]) OR (Systematic Review[pt]) OR (Meta-analysis[pt]) OR (Network Meta-Analysis[pt]) |
| #25 | (“sub-study”[ti]) OR (“sub-analysis”[ti]) OR (“post hoc”[ti]) |
| #26 | #20 AND #21 NOT #22 NOT #23 NOT #24 NOT #25 |
| #27 | #8 AND #14 AND #19 AND #26 |

Supplemental Table 2b. Cochrane Trials search strategy

Data collection period: 1998 to November 1, 2023

Search date: November 1, 2023

No language restrictions

| # | Query |
| --- | --- |
| #1 | [mh “Myocardial ischemia”] OR (“ischemic heart disease” OR “chronic coronary syndrome” OR “angina pectoris” OR “stable angina pectoris” OR “stable angina” OR “unstable angina” OR “unstable angina pectoris” OR “acute coronary syndrome” OR “acute myocardial infarction” OR “myocardial infarction” OR “ST-elevation Myocardial Infarction” OR “ST-segment elevation myocardial infarction” OR “Non-ST-elevation myocardial infarction” OR “Non-ST-segment elevation myocardial infarction”):ti,ab |
| #2 | [mh “Dual Antiplatelet Therapy”] OR (“dual antiplatelet therapy”):ti,ab |
| #3 | [mh “Clopidogrel”] OR (“clopidogrel”):ti,ab OR [mh “Prasugrel Hydrochloride”] OR (“prasugrel”):ti,ab OR [mh “Ticagrelor”] OR (“ticagrelor”):ti,ab OR [mh “Aspirin”] OR (“aspirin”):ti,ab |
| #4 | [mh “Death”] OR (“death” OR “mortality”):ti,ab OR [mh “Cerebrovascular disorders”] OR (“stroke” OR “cerebral infarction”):ti,ab OR [mh “Coronary Thrombosis”] OR (“coronary thrombosis”):ti,ab OR [mh “Coronary Thrombosis”] OR (“coronary thrombosis”):ti,ab OR (“stent thrombosis”):ti,ab OR [mh “Hemorrhage”] OR (“hemorrhage” OR “bleeding”):ti,ab |
| #5 | [mh “Randomized Controlled Trial”] OR (“randomized controlled trial” OR “randomized trial” OR “randomized”):ti,ab |
| #6 | [mh “Vascular Grafting”] OR (graft):ti OR (bypass):ti OR [mh “Anticoagulants”] OR (anticoagu*):ti,ab OR (edoxaban):ti,ab OR (rivaroxaban):ti,ab OR (apixaban):ti,ab OR (dabigatran):ti,ab OR (warfarin):ti,ab OR (cilostazol):ti,ab |
| #7 | [mh “Systematic Review”] OR [mh “Review”] OR [mh “Meta-Analysis”] OR [mh “Network Meta-Analysis”] OR (“Systematic Review” OR “Review” OR “Meta-Analysis” OR “Network Meta-Analysis”):ti |
| #8 | (“sub-study”):ti,ab OR (“sub-analysis”):ti,ab OR (“post hoc”):ti,ab |
| #9 | #1 AND #2 AND #3 AND #4 AND #5 NOT #6 NOT #7 NOT #8 |

Supplemental Table 2c. Scopus search strategy

Data collection period: 1998 to November 1, 2023

Search date: November 1, 2023

No language restrictions

| # | Query |
| --- | --- |
| #1 | “Myocardial ischemia” OR “ischemic heart disease” OR “chronic coronary syndrome” OR “angina pectoris” OR “stable angina pectoris” OR “stable angina” OR “unstable angina” OR “unstable angina pectoris” OR “acute coronary syndrome” OR “acute myocardial infarction” OR “myocardial infarction” OR “ST-elevation Myocardial Infarction” OR “ST-segment elevation myocardial infarction” OR “Non-ST-elevation myocardial infarction” OR “Non-ST-segment elevation myocardial infarction” |
| #2 | “dual antiplatelet therapy” OR “DAPT” |
| #3 | “clopidogrel” OR “prasugrel” OR “ticagrelor” OR “aspirin” |
| #4 | “death” OR “mortality” OR “Cerebrovascular disorders” OR “stroke” OR “cerebral infarction” OR “coronary thrombosis” OR “stent thrombosis” OR “hemorrhage” OR “bleeding” |
| #5 | “randomized controlled trial” OR “randomized trial” OR “randomized” |
| #6 | anticoagu* OR edoxaban OR rivaroxaban OR apixaban OR dabigatran OR warfarin OR cilostazol |
| #7 | “coronary artery bypass grafting” OR “bypass” OR “graft*” |
| #8 | “Systematic Review” OR “Meta-Analysis” OR “Network Meta-Analysis” |
| #9 | “sub-study” OR “sub-analysis” OR “post hoc” |
| #10 | #1 AND #2 AND #3 AND #4 AND #5 NOT #6 NOT #7 NOT #8 NOT #9 |

Supplemental Table 3a. Definitions of trial-defined MACE

| Trial | Definition |
| --- | --- |
| TICAKORIA | Composite endpoint of death from cardiovascular (CV) causes, nonfatal myocardial infarction (MI), or nonfatal stroke |
| ISAR REACT 5 | Composite endpoint of death, MI, or stroke at 1 year after randomization |
| Elderly ACS 2 | Composite endpoint of all-cause mortality, MI, and stroke |
| PRAGUE 18 | The combined occurrence of CV death, nonfatal MI, or stroke |
| Tang et al. | Composite endpoint of overall death, MI, unplanned revascularization, and stroke |
| PHILO | First occurrence of MI, stroke, or death from vascular causes |
| PRASFIT-Elective | Composite endpoint of CV death, nonfatal MI, and nonfatal ischemic stroke |
| PRASFIT-ACS | Composite endpoint of CV death, nonfatal MI, and nonfatal ischemic stroke |
| TRILOGY ACS | Composite endpoint of death from CV causes, nonfatal MI, or nonfatal stroke |
| PLATO | Composite endpoint of death from vascular causes, MI, or stroke |
| Wang et al. | Composite endpoint of MI, stroke, or CV death |
| TRITON-TIMI 38 | Composite endpoint of CV death, nonfatal MI, or nonfatal stroke |
| DISPERSE-2 | Composite incidence of MI (including silent MI), death, stroke, and severe recurrent ischemia |

CV = cardiovascular; MI = myocardial infarction

Supplemental Table 3b. Definitions of trial-defined bleeding events

| Trial | Definition |
| --- | --- |
| TICAKORIA | The primary safety end point was the occurrence of clinically significant bleeding (a composite of major bleeding or minor bleeding according to the PLATO criteria at 12 months. Secondary safety end points included major, minor, or fatal bleeding defined by the PLATO criteria; major or minor bleeding according to the TIMI definition; bleeding according to the BARC definitions; and premature discontinuation of the study medications. |
| ISAR REACT 5 | Incidence of bleeding at 1 year (type 3, 4, or 5 on the BARC scale, which ranges from 0 to 5, with higher values indicating more severe bleeding). |
| Elderly ACS 2 | Bleeding is to be classified according to the BARC scale. Type 2 and 3 bleeding will be considered in the primary end point. |
| PRAGUE 18 | Bleeding defined according to TIMI and BARC criteria. |
| Tang et al. | Incidence of bleeding events as defined by the TIMI criteria. |
| PHILO | Major life-threatening bleeding was defined as fatal bleeding, intracranial bleeding, intrapericardial bleeding with cardiac tamponade, hypovolemic shock or severe hypotension due to bleeding and requiring vasopressors or surgery, decline in Hb ≥5.0 g/dl, or the need for transfusion of at least 4 units of whole blood or packed red blood cells. Bleeding that required medical intervention but did not meet the criteria for major bleeding was defined as minor, and bleeding that did not require intervention was defined as minimal. |
| PRASFIT-Elective | Non-CABG-related TIMI major bleeding. |
| PRASFIT-ACS | Non-CABG-related TIMI major bleeding. |
| TRILOGY ACS | Based on the GUSTO criteria for severe or life-threatening bleeding not related to CABG and TIMI criteria for major bleeding not related to CABG. |
| PLATO | We defined major life-threatening bleeding as fatal bleeding, intracranial bleeding, intrapericardial bleeding with cardiac tamponade, hypovolemic shock or severe hypotension due to bleeding and requiring pressors or surgery, a decline in the Hb level of 5.0 g per deciliter or more, or the need for transfusion of at least 4 units of red cells. We defined other major bleeding as bleeding that led to clinically significant disability (e.g., intraocular bleeding with permanent vision loss) or bleeding either associated with a drop in the Hb level of at least 3.0 g per deciliter but less than 5.0 g per deciliter or requiring transfusion of 2 to 3 units of red cells. |
| Wang et al. | PLATO major bleeding. |
| TRITON-TIMI 38 | Major safety end points include TIMI major bleeding, TIMI major plus minor bleeding, and life-threatening bleeding. |
| DISPERSE-2 | Major bleeding—fatal/life-threatening” (based on the TIMI major bleeding category) was defined as any 1 of the following: fatal, intracranial, intrapericardial with cardiac tamponade, bleeding leading to hypovolemic shock or severe hypotension or requiring pressors or surgery, clinically overt or apparent bleeding associated with a decrease in Hb of >5 g/dl (3.1 mmol/l), or bleeding associated with transfusion of 4 or more units. To account for transfusions, Hb measurements were adjusted for any packed red blood cell or whole blood transfusions given between 2 blood measurements; a transfusion of 1 U of blood was assumed to result in an increase of 1 g/dl in Hb. “Major bleeding—other” (based on the TIMI minor bleeding category) was defined as any 1 of the following: significantly disabling bleeding (e.g., intraocular with permanent vision loss), clinically overt or apparent bleeding associated with a decrease in Hb of 3 to 5 g/dl (1.9 to 3.1 mmol/l), or transfusion of 2 to 3 U (whole blood or packed red blood cells) for bleeding. “Minor bleeding” was defined as any 1 of the following: bleeding that required medical intervention (e.g., epistaxis requiring visit to medical facility for packing) or transfusion of 1 U (whole blood or packed red blood cells). “Minimal bleeding” was defined as any other reported episode of bleeding that did not meet the above criteria (e.g., bruising, bleeding gums, oozing from injection sites). The Independent Clinical Adjudication Committee also prospectively classified bleeding according to the definitions used in the CURE trial, as well as TIMI and GUSTO (Global Utilization of Streptokinase and Tissue plasminogen activator for Occluded coronary arteries) criteria. |

PLATO = Platelet Inhibition and Patient Outcomes; TIMI = Thrombolysis In Myocardial Infarction; BARC = Bleeding Academic Research Consortium; CABG = coronary artery bypass graft; Hb = hemoglobin; GUSTO = Global Use of Strategies to Open Occluded Coronary Arteries; CURE = Clopidogrel in Unstable angina to prevent Recurrent ischemic Events; Hb = hemoglobin

Supplemental Table 3c. Definitions of trial-defined death

| Trial | Definition |
| --- | --- |
| TICAKORIA | Death was considered to have a CV cause unless an unequivocal, non-CV cause could be established. |
| ISAR REACT 5 | The primary end point includes death from any cause. In addition, the cause of death will be adjudicated. If autopsy has been performed autopsy reports should be solicited for determination of cause of death.  Cardiac death:  Any death due to proximate cardiac cause (e.g. myocardial infarction, low-output failure, fatal arrhythmia), unwitnessed death and death of unknown cause, and all procedure-related deaths, including those related to concomitant treatment, will be classified as cardiac death.  Vascular death:  Death caused by noncoronary vascular causes, such as cerebrovascular disease, pulmonary embolism, ruptured aortic aneurysm, dissecting aneurysm, or other vascular diseases.  Noncardiovascular death:  Any death not covered by the above definitions, such as death caused by infection, malignancy, sepsis, pulmonary causes, accident, suicide, or trauma.  All deaths are considered cardiac unless an unequivocal noncardiac cause can be established. Specifically, any unexpected death even in patients with coexisting potentially fatal noncardiac disease (e.g. cancer, infection) should be classified as cardiac. |
| Elderly ACS 2 | All-cause mortality will be considered for the primary analysis. For secondary analysis, all deaths will be assumed cardiovascular in nature unless a non–cardiovascular cause can be clearly shown. |
| PRAGUE 18 | Death was defined as a summary of death from any cause. Cardiovascular death was defined as any death for a demonstrable cardiovascular cause, or any death that was not clearly attributable to a non-cardiovascular cause. |
| Tang et al. | NA |
| PHILO | Defined as in the PLATO study. |
| PRASFIT-Elective | Cardiovascular death was defined as any cardiovascular-related death. |
| PRASFIT-ACS | Cardiovascular death was defined as any death attributable to a cardiovascular event. |
| TRILOGY ACS | Death from documented cardiovascular cause or not clearly attributable to noncardiovascular causes. |
| PLATO | Death from vascular causes was defined as death from cardiovascular causes or cerebrovascular causes and any death without another known cause. |
| Wang et al. | All variables were determined as defined in the PLATO trial. |
| TRITON-TIMI 38 | Cardiovascular death is considered as any death with a demonstrable CV cause or any death that is not clearly attributable to a non-CV cause. |
| DISPERSE-2 | NA |

CV = cardiovascular; PLATO = Platelet Inhibition and Patient Outcomes

Supplemental Table 3d. Definitions of trial-defined MI

| Trial | Definition |
| --- | --- |
| TICAKORIA | The diagnosis of MI was based on the Third Universal Definition of Myocardial Infarction. |
| ISAR REACT 5 | The definition of myocardial infarction used in this trial is adapted from the Third Universal Definition of Myocardial Infarction. Cardiac troponin will be used as the preferred biomarker. CK-MB (and CK) values will be assessed concurrently and used in the case that troponin values are not available.  1. Spontaneous myocardial infarction, not related to PCI or CABG Detection of a rise and/or fall in cardiac biomarkers (preferebly cardiac troponin) with at least one value above the 99th percentile upper reference limit (URL) and with at least one of the following  - symptoms of ischemia  - development of pathological Q waves in the ECG  - new or presumed new ST-segment  - T-wave changes (ST-T changes) or new left bundle branch block  - imaging evidence of new loss of viable myocardium or new regional wall motion abnormality  2. Myocardial infarction after randomization and before PCI  Recurrent symptoms of cardiac ischemia or hemodynamic instability plus one of the following criteria  - new or presumed new ST-segment elevation or new left bundle branch block distinct from the last ECG or  - in patients with normal biomarkers and not presenting with ST-segment elevation MI on admission: detection of a rise and/or fall in cardiac biomarkers (preferably cardiac troponin) with at least one value above the 99th percentile upper reference limit (URL)  - if the baseline Troponin values are elevated and are stable or falling, then a rise of >20% is required  - development of new pathological Q waves in the ECG distinct from the coronary territory identified on admission  - imaging evidence of new loss of viable myocardium or new regional wall motion abnormality  3. PCI-related myocardial infarction (within 48 h after PCI)  Cardiac enzymes (Troponin T or I, CK and CK-MB) will be determined on admission, 4-6 hours after admission (before angiography, in all patients with non-urgent PCI) and from blood drawn from the arterial sheath in the cath lab immediately after sheath insertion and before PCI. Biomarker course will be used for redefinition of baseline status in patients with NSTE-ACS, i.e. to differentiate unstable angina pectoris from NSTEMI and to better describe biomarker course in NSTEMI patients. Based on the 2 sets of biomarkers the baseline status will be redefined:  - If biomarkers on admission have been normal (initial diagnosis of unstable angina) and biomarkers are rising > 99th percentile URL in the second sample (before catherization or from the arterial sheath) without recurrent symptoms of ischemia the initial diagnosis of unstable angina is revised to NSTEMI on admission.  - If biomarkers on admission have been elevated (diagnosis of NSTEMI) then it will be documented whether biomarker values are stable, rising or falling. |
| Elderly ACS 2 | MI is defined by a consistent clinical scenario, confirmed by the presence of either ECG evidence or cardiac marker evidence. ECG evidence of MI requires new Q waves (>0.04 s) in 2 or more contiguous leads which is not an ambiguous change from baseline. Cardiac marker evidence of MI requires either troponin or CK-MB elevation greater than the ULN. After PCI, CK-MB elevation must be >3× ULN and increased by at least 50% over the previous value. In the 72-h period after CABG, the definition for an MI requires both enzyme and ECG criteria if the CK-MB is >5× ULN but <10× ULN. If the cardiac markers are >10× ULN, ECG criteria are not required. |
| PRAGUE 18 | Non-fatal myocardial infarction was required to be distinct from the index event and was defined according to the Third Universal Definition of Myocardial Infarction. |
| Tang et al. | MI was defined according to the guidelines of the European Society of Cardiology. |
| PHILO | Defined as in the PLATO study. |
| PRASFIT-Elective | Non-fatal MI was defined as any of the following 3 events.  1. In patients with normal creatine kinase (CK)-MB before PCI/CABG, CK-MB had to be (1) ≥3-fold the upper limit of normal (ULN) in 2 samples or ≥5-fold the ULN in 1 sample obtained <48 h after PCI, or (2) ≥10-fold the ULN in 1 sample obtained <48 h after CABG. Patients whose CK-MB exceeded ULN before PCI/CABG had to show a transient decrease with a subsequent increase ≥1.5-fold the previous value and satisfy (1) or (2).  2. More than 48 h after PCI, CK-MB or troponin had to be ≥2-fold the ULN, accompanied by 1 or more of the following: new or recurrent sustained ischemic chest pain, hemodynamic decompensation, or new or recurrent ST elevation/depression ≥0.1 mV.  3. Abnormal Q waves had to persist for ≥0.04 s. |
| PRASFIT-ACS | Nonfatal MI was defined as events fulfilling at least one of the following 3 criteria.  (1) In patients with normal creatine kinase (CK)-MB before PCI/coronary artery bypass grafting (CABG), CK-MB had to be (a) ≥3-fold the upper limit of normal (ULN) in 2 samples obtained, or ≥5-fold the ULN in 1 sample obtained <48 h after PCI, or (b) ≥10-fold the ULN in 1 sample obtained <48 h after CABG. Patients whose CK-MB exceeded the ULN before PCI/CABG had to show a transient decrease with a subsequent increase of ≥1.5-fold the previous value and satisfy (a) or (b).  (2) More than 48 h after PCI, the CK-MB or troponin levels had to be ≥2-fold the ULN, accompanied by 1 or more of the following: new or recurrent sustained ischemic chest pain, hemodynamic decompensation, or new or recurrent ST elevation/depression ≥0.1 mV.  (3) Abnormal Q waves had to persist for ≥0.04 s. |
| TRILOGY ACS | Elevation or re-elevation of the ST-segment; either ischemic symptoms ≥ 20 min or hemodynamic decompensation. Troponin (T or I) or CK-MB > the ULN; either ischemic symptoms ≥20 min or ST-segment deviation ≥1 mm in ≥1 lead. MI associated with revascularization. New Q waves ≥0.04 s or pathology distinct from the index event. |
| PLATO | Myocardial infarction was defined in accordance with the universal definition proposed in 2007. |
| Wang et al. | All variables were determined as defined in the PLATO trial. |
| TRITON-TIMI 38 | Myocardial infarction must be distinct from the index event and is defined by symptoms suggestive of ischemia/infarction, electrocardiographic data, cardiac biomarker, or pathologic evidence of infarction dependent on the clinical situation using criteria adapted from the definition developed by the American College of Cardiology. |
| DISPERSE-2 | Defined as troponin (T or I) or CK-MB elevation greater than the local MI decision limit, or if these markers were not available, total CK greater than twice the local MI decision limit or electrocardiographic evidence of ischemia, defined as the presence of new or presumably new ST-segment depression ≥0.5 mm (0.05 mV), transient ST-segment elevation ≥1 mm (0.1 mV), or T-wave inversion ≥1 mm (0.1 mV) in 2 or more contiguous leads. |

MI = myocardial infarction; CK = creatine kinase; URL = upper reference limit; ECG = electrocardiography; PCI = percutaneous coronary intervention; NSTE-ACS = non-ST-segment elevation acute coronary syndrome; NSTEMI = non-ST-segment elevation myocardial infarction; PLATO = Platelet Inhibition and Patient Outcomes; ULN = upper limit of normal; CABG = coronary artery bypass graft

Supplemental Table 3e. Definitions of trial-defined stroke

| Trial | Definition |
| --- | --- |
| TICAKORIA | Stroke was defined as a focal loss of neurologic function caused by an ischemic or hemorrhagic event, with residual symptoms lasting at least 24 h or leading to death. |
| ISAR REACT 5 | Stroke is defined as the new onset of focal or global neurological deficit caused by ischemia or hemorrhage within or around the brain and lasting for more than 24 hours or leading to death.  The diagnosis of stroke requires confirmation by CT, MRI or autopsy.  Etiology:  - intracranial hemorrhage will be diagnosed by appropriate brain imaging (e.g. CT or MRI) that shows the presence of an acute blood mass.  - ischemic or bland infarction will be diagnosed by appropriate imaging which shows the presence of one or more of the following: hypodensity, edema, midline shift, or ventricular effacement without evidence of hemorrhage.  - hemorrhagic conversion of an ischemic infarction will be diagnosed by appropriate imaging that shows an ischemic infarction with localized petechial or confluent bleeding into necrotic tissue.  Degree of severity:  Non disabling stroke:  - If he or she had no sequels or only a minor deficit (with the functional status unchanged). Modified Rankin scale grade of ≤ 3.  Disabling stroke:  - If at the time of hospital discharge he or she had a moderate deficit (substantial limitation of activity and capabilities) or a severe deficit (inability to live independently or work). Modified Rankin scale grade of ≥ 4. |
| Elderly ACS 2 | Any new neurologic deficit lasting >24 h. CT or MRI is required to confirm the stroke and to distinguish between ischemic and hemorrhagic stroke. Strokes will be also classified as fatal or nonfatal: nonfatal stroke will be also classified as disabling and nondisabling. Fatal and disabling stroke will be counted in the primary end point of the study, whereas also nondisabling strokes will be counted in the secondary analyses. |
| PRAGUE 18 | Stroke was defined as a rapid onset of a new neurological deficit caused by an ischemic or hemorrhagic central nervous system event with symptoms lasting at least 24 hours from their onset or leading to death. |
| Tang et al. | Stroke was defined as a focal loss of neurologic function caused by either an ischemic or a hemorrhagic event. |
| PHILO | Defined as in the PLATO study. |
| PRASFIT-Elective | The presence of neurological symptoms or signs consistent with stroke and confirmation on magnetic resonance imaging. |
| PRASFIT-ACS | The presence of neurological symptoms or signs consistent with stroke and confirmation by magnetic resonance imaging. |
| TRILOGY ACS | Rapid onset of new, persistent neurologic deficit lasting >24 h: “ischemic” or “hemorrhagic” (if imaging data available), or “uncertain cause” (if imaging data unavailable). |
| PLATO | Stroke was defined as focal loss of neurologic function caused by an ischemic or hemorrhagic event, with residual symptoms lasting at least 24 hours or leading to death. |
| Wang et al. | All variables were determined as defined in the PLATO trial. |
| TRITON-TIMI 38 | Stroke is defined as the rapid onset of new, persistent, neurologic deficit lasting at least 24 hours (or resulting in death before 24 hours). |
| DISPERSE-2 | NA |

CT = computed tomography; MRI = magnetic resonance imaging; PLATO = Platelet Inhibition and Patient Outcomes

Supplemental Table 4a. Criteria for inclusion and exclusion in individual trials

| Inclusion criteria | Exclusion criteria |
| --- | --- |
| TICAKORIA, Park et al. 2019 | |
| 1. Male and female patients ≥18 years of age.  2. Index event of non-ST or ST segment elevation ACS, with an onset of symptoms during the previous 24 hours, for whom invasive management is planned, defined by:  For patients who had an ACS without ST-segment elevation, at least two of the following three criteria had to be met:  ST-segment changes on electrocardiography (ST-segment depression or transient elevation ≥ 1 mm in two or more 2 contiguous leads), indicating ischemia;  A positive test of a biomarker (Troponin I or T or CK-MB greater than the upper limit of normal), indicating myocardial necrosis; or  One of several risk factors (age ≥60 years; previous MI or coronary-artery bypass grafting; coronary artery disease with stenosis of ≥50% in at least two vessels; previous ischemic stroke, transient ischemic attack, carotid stenosis of at least 50%, or cerebral revascularization; diabetes mellitus; peripheral arterial disease; or chronic renal dysfunction, defined as a creatinine clearance of <60 ml/minute/1.73 m^2^ of body surface area).  For patients who had an ACS with ST-segment elevation, the following two inclusion criteria had to be met:  Persistent ST-segment elevation of at least 1 mm in at least two contiguous leads or  A new left bundle-branch block, and the intention to perform primary PCI.  3. Written informed consent prior to any study specific procedures. | 1. Hypersensitivity to ticagrelor or aspirin.  2. Need for chronic oral anticoagulant therapy or chronic low-molecular-weight heparin or long-term treatment with fondaparinux.  3. Fibrinolytic therapy within 24 hours before randomization.  4. Any condition which in the opinion of the investigator would make it unsafe or unsuitable for the patient to participate in this study (e.g., active malignancy other than squamous cell or　basal cell skin cancer, use of strong or moderate CYP2C19 inhibitors, including omeprazole and esomeprazole, long-term concomitant treatment with non-steroidal anti-inflammatory drugs).  5. Concomitant oral or intravenous therapy with strong CYP3A inhibitors (ketoconazole, itraconazole, voriconazole, telithromycin, clarithromycin, nefazodone, ritonavir, saquinavir, nelfinavir, indinavir, atazanavir, grapefruit juice >1 L/day), CYP3A substrates with narrow therapeutic indices (cyclosporine, quinidine), or strong CYP3A inducers (rifampin/rifampicin, phenytoin, carbamazepine).  6. Life expectancy <6 months based on investigator’s judgment.  7. Dementia likely to jeopardize understanding of information pertinent to study conduct or compliance to study procedures.  8. Severe hypertension that may put the patient at risk.  9. Patients considered to be at risk of bradycardic events (e.g., known sick sinus syndrome or second or third degree atrioventricular block) unless already treated with a permanent pacemaker.  10. Known severe liver disease (e.g., ascites and/or clinical signs of coagulopathy).  11. Renal failure requiring dialysis.  12. A known bleeding diathesis, hemostatic or coagulation disorder, or systemic bleeding, whether resolved or ongoing.  13. History of previous intracranial bleeding at any time, gastrointestinal bleeding within the past 6 months, or major surgery within 30 days (if the surgical wound is judged to be associated with an increased risk of bleeding).  14. History of thrombocytopenia or neutropenia.  15. Women of child-bearing potential (i.e., those who were not chemically or surgically sterilized or who were not postmenopausal) who were not willing to use a medically accepted method of contraception considered reliable in the judgment of the investigator or women with a positive pregnancy test at first visit.  16. Concern for inability of the patient to comply with study procedures and/or follow-up (e.g., alcohol or drug abuse).  17. Involvement in the planning and/or conduct of another study. |
| ISAR REACT 5, Schupke et al. 2019 | |
| - Hospitalization for an ACS (unstable angina pectoris, NSTEMI, or STEMI) with planned invasive strategy  STEMI  Chest discomfort suggestive of cardiac ischemia ≥ 20 minutes at rest, within 24 h prior to randomization with 1 of the following ECG features:  - ST-segment elevation ≥ 1 mm in ≥ 2 contiguous ECG leads or  - new or presumably new left bundle branch block (LBBB)  Non-ST-segment elevation ACS (unstable angina or NSTEMI)  Chest discomfort suggestive of cardiac ischemia for ≥ 10 minutes at rest within 48 h prior to randomization + 1 of the following criteria:  - ST-segment depression ≥ 1 mm in ≥ 1 or 2 contiguous ECG leads or  - Troponin T or I or CK-MB greater than the upper limit of normal or  - 2 of the following clinical criteria:  Age ≥ 60 years  ≥ 3 risk factors for coronary artery disease (arterial hypertension, hypercholesterolemia, family history, diabetes mellitus, current smoker)  Diabetes mellitus  Aspirin use in the past 7 days  Severe angina (≥ 2 episodes within the last 24 hours)  Chronic renal dysfunction  Prior MI or CABG  Known CAD with ≥ 50% stenosis in ≥ 2 vessels  Carotid stenosis ≥ 50% or cerebral revascularization  Peripheral artery disease  age ≥ 18 years | - intolerance of or allergy to ticagrelor or prasugrel.  - history of any stroke, transient ischemic attack or intracranial bleeding.  - known intracranial neoplasm, intracranial arteriovenous malformation or intracranial aneurysm.  - active bleeding, clinical findings, that in the judgement of the investigator are associated with an increased risk of bleeding.  - fibrin-specific fibrinolytic therapy less than 24 h before randomization, non-fibrinspecific fibrinolytic therapy less than 48 h before randomization.  - known platelet count < 100.000/μL at the time of screening.  - known anemia (Hb < 10 g/dL) at the time of screening.  - oral anticoagulation that cannot be safely discontinued for the duration of the study.  - INR known to be greater than 1.5 at the time of screening.  - chronic renal insufficiency requiring dialysis.  - moderate or severe hepatic dysfunction (Child Pugh B or C).  - increased risk of bradycardia events (Sick Sinus, AV block grade II or III, bradycardia-induced syncope).  - index event is an acute complication (< 30 days) of PCI.  - concomitant medical illness that in the opinion of the investigator is associated with a life expectancy < 1 year.  - concomitant oral or i.v. therapy with strong CYP3A inhibitors (e.g. ketoconazole, itraconazole, voriconazole, telithromycin, clarithromycin, nefazodone, ritonavir, saquinavir, nelfinavir, indinavir, atazanavir, grapefruit juice >1 L/d), CYP3A substrates with narrow therapeutic indices (e.g. cyclosporine, quinidine), or strong CYP3A inducers (e.g. rifampin/rifampicin, phenytoin, carbamazepine, dexamethason, phenobarbital) that cannot be safely discontinued  - ≥ 1 doses of ticagrelor or prasugrel within 5 days before randomization  - no written informed consent  - participation in another investigational drug study  - previous enrolment in this study  - for women of childbearing potential no negative pregnancy test and no agree to use a reliable method of birth control during the study.  - pregnancy, giving birth within the last 90 days, or lactation.  - inability to cooperate with protocol requirements. |
| Elderly ACS 2, Savonitto et al. 2018 | |
| We enrolled patients >74 years of age with ST-segment–elevation (STE)— or NSTE-ACS treated with PCI during index admission. To be eligible, patients with NSTE-ACS had to show at least 1 of the following characteristics: elevated troponin levels, diabetes mellitus, prior MI, ≥1 new ischemic episode while on standard treatment during the index hospitalization, or stent thrombosis. | We excluded patients with a history of stroke, gastrointestinal or genitourinary bleeding of clinical significance within the previous 6 weeks, hemoglobin level on admission <10 g/dL unless this was considered to be secondary to renal dysfunction or known myelodysplasia, platelet count <90 000 cells/mL, secondary causes of ischemia, ongoing oral anticoagulant treatment or a spontaneous international normalized ratio >1.5 at the time of screening, concomitant severe obstructive lung disease, malignancy, or neurological deficit limiting follow-up or adherence to the study protocol. Patients unable to give at least verbal informed consent to the study or already under treatment with prasugrel or ticagrelor were also excluded. |
| PRAGUE 18, Motovska et al. 2018 | |
| Patients with AMI treated with a primary (more general term used for both STEMI and very-high-risk non-STEMI) PCI strategy were enrolled in the study. The study inclusion criteria were the following: AMI indicated for emergent (within 120 minutes of admission to a cardiac center) coronary angiography with or without PCI and a signed informed consent. Hemodynamic instability was not an exclusion criterion for study participation. A diagnosis of AMI was determined from the clinical presentation and an ECG finding of ST-segment elevation on 2 related leads at a minimum by >1 mm, ST-segment depression on 3 leads at a minimum by >2 mm, or a new bundle-branch block. | history of stroke, serious bleeding within the previous 6 months, indication for chronic oral anticoagulation therapy, administration of clopidogrel ≥300 mg or of any other antiplatelet medication before randomization (with the exception of aspirin and a lower dose of clopidogrel), patients older than 75 years whose body weight was also <60 kg (i.e., the presence of both parameters simultaneously was an exclusion criterion), moderate or severe hepatic dysfunction, concomitant treatment with a strong CYP3A4 inhibitor, or known hypersensitivity to prasugrel or ticagrelor. |
| Tang et al., 2016 | |
| We enrolled patients diagnosed with STEMI at the Zhujiang Hospital of Southern Medical University and the First Hospital of Qinhuangdao, China, between January 1, 2013 and April 30, 2015. The inclusion criteria were as follows: (1) age, >18 years; (2) chest discomfort for >20 minutes and no response to nitroglycerin; (3) time from the onset of symptoms to randomization <12 hours; (4) eligible for PPCI; (5) ST-segment elevation of >1 mm in 2 or more limb leads or >2 mm in 2 or more contiguous precordial leads; (6) Killip class of ≤3; and (7) the provision of informed consent. | The exclusion criteria were as follows: (1) cardiogenic shock, defined as systolic blood pressure of <90/60 mm Hg and no response to fluids; (2) thrombolysis within the past 24 hours; (3) oral anticoagulation therapy or current use of P2Y12 antagonists; (4) malignant or life-threatening diseases; (5) contraindications to aspirin, clopidogrel, or ticagrelor; (6) inability to provided informed consent; (7) suspected mechanical complications of STEMI; or (8) CABG within the previous year. |
| PHILO, Goto et al. 2015 | |
| Patients were eligible if they were hospitalized for ST- or non- ST-segment elevation ACS with onset of symptoms during the previous 24 h (cardiac ischemic symptoms of ≥10 min duration at rest) and if PCI was planned. Inclusion and exclusion criteria were similar to those for PLATO. | Any contraindication against the use of clopidogrel; active bleeding or a history of bleeding; fibrinolytic therapy within 24 h before randomization; need for oral anticoagulation therapy; increased risk of bradycardia; and concomitant therapy with a strong CYP3A inhibitor or inducer. The aim was to recruit approximately 800 patients from Japan and East Asian countries. |
| PRASFIT-Elective, Isshiki et al. 2014 | |
| Patients aged ≥20 years who were scheduled for elective PCI to treat CAD such as stable angina or prior MI with stenosis confirmed on coronary computed tomography were eligible for this study. | (1) acute MI, ischemic symptoms (at rest), or unstable angina within 72 h after the onset of these conditions; (2) left main artery disease, chronic complete obstruction, scheduled for stenting to treat venous graft diseases, or scheduled for rotablator treatment; (3) current or history of intracranial bleeding; (4) current or history of cerebral infarction (plus one of the following: needing anticoagulation therapy, age ≥75 years, or within 6 months after the onset of cerebral infarction); (5) blood disorder, such as hemophilia; (6) tendency for bleeding; (7) uncontrolled hypertension (systolic/diastolic pressure: ≥160/≥100 mmHg); (8) severe heart failure (NYHA class III–IV); (9) severe arrhythmia; (10) current or history of thrombotic thrombocytopenic purpura or agranulocytosis; and (11) severe blood, liver, or renal disorder. |
| PRASFIT-ACS, Saito et al. 2014 | |
| The present study was conducted in Japanese ACS patients who satisfied all of the following criteria and were scheduled for coronary artery stenting: males/females aged ≥20 years; presence of chest discomfort or ischemic symptoms lasting ≥10 min within 72 h before randomization; ST-segment deviation ≥1 mm, or T-wave inversion ≥3 mm, or elevated levels of cardiac biomarkers for necrosis. | The major exclusion criteria were: refractory life-threatening ventricular arrhythmia; history or a current diagnosis of thrombocytopenia purpura or agranulocytosis; pancytopenia or aplastic anemia; history of intracranial bleeding; history of ischemic stroke/TIA; history of or predisposition to hemorrhagic disease; poorly controlled hypertension; severe hepatic or renal impairment; NYHA grade IV heart failure; and administration of a thienopyridine drug within 5 days before starting the study drug or a thrombolytic drug within 24 h before starting the study drug. All of the patients provided informed consent before enrollment. The present study was conducted in accordance with the Declaration of Helsinki and Good Clinical Practice, and was approved by the institutional review boards at all 162 participating centers. |
| TRILOGY ACS, Roe et al. 2012 | |
| Main inclusion criteria  1. Chest discomfort or ischemic-equivalent symptoms ≥5 min duration at rest within 24 h of index event  2. ACS based on diagnostic criteria (A) within 10 d of randomization and planned for medical management  3. ≥1 high-risk enrichment criterion (B)  4. Age ≥18 y  A. Disease diagnostic criteria  1. Unstable angina: ST-segment depression >1 mm in ≥2 ECG leads without elevated troponin (T or I) or CK-MB  2. Non–ST-segment elevation MI: troponin (T or I) or CK-MB >ULN (defined by local laboratory assay), or total CK ≥2× ULN if troponin or CK-MB unavailable, with no persistent ST-segment elevation  B. High-risk enrichment criteria (derived from GRACE risk score)  1. Age ≥60 y  2. Prior MI  3. Diabetes mellitus (treated with oral medications and/or insulin)  4. PCI or CABG ≥30 d before index event | Cardiovascular  1. Medical management decision >72 h after index event without commercial clopidogrel treatment within 72 h of index event  2. Planned PCI or CABG for index event  3. PCI or CABG performed within previous 30 d  4. STEMI as index event  5. NYHA class IV heart failure, cardiogenic shock, or refractory ventricular arrhythmias within previous 24 h  6. Absence of at least 1 stenosis (estimated to be ≥30%) in any native coronary artery if angiography performed for index event (not applicable to patients with prior PCI/CABG)  Bleeding  1. Received fibrinolytic therapy  2. Any of the following:  –Prior ischemic or hemorrhagic stroke  –Intracranial neoplasm, arteriovenous malformation, or aneurysm  –Prior transient ischemic attack  3. Bleeding diathesis or clinical findings associated with an unacceptably high bleeding risk  4. INR >1.5 (if performed)  5. Platelet count <100 000/mm^3^  6. Hb <10 g/dL  7. Currently receiving hemodialysis or peritoneal dialysis  Concomitant medication  1. Intolerance/allergy to aspirin or thienopyridines  2. Received ticlopidine within 5 d of randomization  3. Receiving prasugrel at time of screening  4. Receiving oral anticoagulants or anticipated to require oral anticoagulants during the study  5. Daily NSAIDs or COX-2 inhibitors that cannot be discontinued, or anticipated to require >2 week of daily treatment during study |
| PLATO, Wallentin et al. 2009 | |
| Hospitalized for potential ST-segment elevation or non–ST-segment elevation ACS, with onset during the previous 24 hours, documented by cardiac ischemic symptoms due to atherosclerosis of ≥10 minutes' duration at rest, ≥18 years of age, not pregnant, and with informed consent.  And: ≥2 of the following:  1. ST-segment changes on ECG indicating ischemia. ST-segment depression or transient elevation ≥ 1 mm in two or more 2 contiguous leads  2. Positive biomarker indicating myocardial necrosis. Troponin I or T or CK-MB greater than the upper limit of normal  3. One of the following:  (a) ≥60 y of age  (b) Previous MI or CABG  (c) CAD with ≥50% stenosis in ≥2 vessels  (d) Previous ischemic stroke, TIA (hospital-based diagnosis), carotid stenosis (≥50%), or cerebral revascularization  (e) Diabetes mellitus  (f) Peripheral artery disease  (g) Chronic renal dysfunction  Or:  Persistent ST-segment elevation ≥1 mm (not known to be preexisting or due to a coexisting disorder) in ≥2 contiguous leads or new LBBB plus primary PCI planned. | Drug related  1. Contraindication to clopidogrel or other reason that study drug should not be administered (eg, hypersensitivity, moderate or severe liver disease, active bleeding or bleeding history, major surgery within 30 d)  2. Oral anticoagulation therapy that cannot be stopped  3. Fibrinolytic therapy planned or within the previous 24 h  4. Concomitant oral or IV therapy with strong CYP3A inhibitors (ketoconazole, itraconazole, voriconazole, telithromycin, clarithromycin, nefazodone, ritonavir, saquinavir, nelfinavir, indinavir, atazanavir, grapefruit juice >1 L/d), CYP3A substrates with narrow therapeutic indices (cyclosporine, quinidine), or strong CYP3A inducers (rifampin/rifampicin, phenytoin, carbamazepine)  Treatment related  1. Index event is an acute complication of PCI  2. PCI after index event and before first study dose  Medical  1. Increased risk of bradycardiac events  2. Dialysis required  3. Known clinically important thrombocytopenia⁎  4. Known clinically important anemia  5. Any other condition that may put the patient at risk or influence study results in the investigators' opinion (eg, cardiogenic shock, severe hemodynamic instability, active cancer)  General  1. Participant in another investigational drug or device study within 30 d  2. Pregnancy or lactation  3. Any condition that increases the risk for noncompliance or being lost to follow-up  4. Involvement in the planning or conduct of the study  5. Previous enrollment or randomization in this study |
| Wang et al., 2016 | |
| Patients were included with the diagnosis of ACS made according to the European Society of Cardiology guideline. | 1) had any contraindication against the use of P2Y12 inhibitors, 2) were under DAPT, anticoagulation, and fibrinolytic therapy, 3) had active bleeding or increased bleeding risk such as malignancy, surgery, trauma, fracture, or organ biopsy, 4) had clinically significant out-of-range values for platelet count or Hb, and 5) had renal function failure requiring dialysis, hypertension with systolic blood pressure >180 mmHg or diastolic blood pressure >110 mmHg, or cardiogenic shock with systolic blood pressure ,<80 mmHg lasting for >30 minutes. |
| TRITON-TIMI 38, Wiviott et al. 2007 | |
| (1) ACS based on the disease diagnostic criteria with planned PCI  (2) Legal age (and ≥18 y) and competent mental condition to provide written informed consent  (3) For women of childbearing potential only, test negative for pregnancy between ACS presentation and enrollment (based on a urine or serum pregnancy test) and agree to use a reliable method of birth control during the study | Cardiovascular exclusion criteria  (1) Cardiogenic shock at the time of randomization  (2) Refractory ventricular arrhythmias  (3) NYHA class IV congestive heart failure Bleeding risk exclusion criteria  (4) Fibrin-specific fibrinolytic therapy less than 24 h before randomization  (5) Non–fibrin-specific fibrinolytic therapy less than 48 h before randomization  (6) Active internal bleeding or history of bleeding diathesis  (7) Clinical findings, in the judgment of the investigator, associated with an increased risk of bleeding  (8) Any of the following:  (a) History of hemorrhagic stroke  (b) Intracranial neoplasm, arteriovenous malformation, or aneurysm  (c) Ischemic stroke within 3 months prior to screening  (9) International normalized ratio known to be greater than 1.5 at the time of screening  (10) Platelet count of less than 100 000/mm^3^ at the time of screening  (11) Anemia (Hb <10 g/dL) at the time of screening Prior/concomitant therapy exclusion criteria  (12) One or more doses of a thienopyridine 5 d or less before PCI  (13) Oral anticoagulation or other antiplatelet therapy that cannot be safely discontinued for the duration of the study  (14) Daily treatment with nonsteroidal antiinflammatory drugs or cyclooxygenase-2 inhibitors General exclusion criteria  (15) Investigative site personnel directly affiliated with the study or immediate family  (16) Employed by Eli Lilly and Company; Ube Industries Limited, Daiichi Sankyo Co.; The TIMI Study Group; Quintiles  (17) Treatment within the last 30 d with an investigational drug or are presently enrolled in another drug or device study  (18) Previously completed or withdrawn from this study or any other study investigating prasugrel  (19) Women who are known to be pregnant, have given birth within the past 90 d, or are breast-feeding  (20) Concomitant medical illness that in the opinion of the investigator is associated with reduced survival over the expected treatment period  (21) Known severe hepatic dysfunction  (22) Any condition associated with poor treatment compliance, including alcoholism, mental illness, or drug dependence  (23) Intolerance of or allergy to aspirin, ticlopidine, or clopidogrel  (24) May be unable to cooperate with protocol requirements and follow-up procedures |
| DISPERSE-2, Cristopher et al. 2007 | |
| Men and women not of childbearing potential were eligible for inclusion if they were hospitalized for NSTE-ACS within the preceding 48 h and were age ≥18 years. Patients had to have experienced ischemic symptoms of ≥10 min duration at rest, with either biochemical marker evidence of MI (defined as troponin T or I, CK-MB elevation greater than the local MI decision limit, or, if these markers were not available, total CK greater than twice the local MI decision limit) or electrocardiographic evidence of ischemia, defined as the presence of new or presumably new ST-segment depression ≥0.5 mm (0.05 mV), transient ST-segment elevation ≥1 mm (0.1 mV), or T-wave inversion ≥1 mm (0.1 mV) in 2 or more contiguous leads. | Exclusion criteria included persistent ST-segment elevation ≥20 min, more than 48 h from onset of symptoms, index event occurring as a consequence of PCI within the prior 48 h or performance of PCI within 48 h before randomization (i.e., patients had to be randomized pre-PCI); angiography showing no significant coronary stenosis; and any of the following conditions associated with increased risk of bleeding: history of intracranial, intraocular, spinal, retroperitoneal, or atraumatic intra-articular bleeding; gastrointestinal bleeding within the prior 6 months; gastric or duodenal ulcer disease verified by endoscopy or radiographic testing within the prior 6 months; persistent uncontrolled hypertension >180/100 mm Hg; any known hemorrhagic disorder; major surgical procedure or trauma within the prior 30 days; or intracranial aneurysm or vascular malformation. Other exclusion criteria included CABG within the 3 months before randomization, nonhemorrhagic stroke within the prior 30 days, active cancer (excluding skin basal cell carcinoma), oral anticoagulation therapy within the prior 7 days or need for chronic oral anticoagulation, chronic daily dosing with nonselective nonsteroidal anti-inflammatory drugs, thrombolytic therapy within the prior 7 days, contraindications for aspirin treatment, concomitant therapy with digoxin or strong cytochrome P450 3A4 inhibitors or cytochrome P450 3A4 substrates with a narrow therapeutic index, known lactose intolerance (due to the excipient in the capsules), serum creatinine level >3.0 mg/dl (265 µmol/l), known active liver disease or elevated liver function tests of alanine aminotransferase >2× the upper limit of normal or total bilirubin >1.5× the upper limit of normal at the local laboratory, Hb level <10 g/dl (6.2 mmol/l), platelet count <100 × 10^9^/l, and participation in another investigational drug study within 1 month before randomization. |

ACS = acute coronary syndrome; CK = creatine kinase; NSTEMI = non-ST-segment elevation myocardial infarction; STEMI = ST-segment elevation myocardial infarction; ECG = electrocardiography; LBBB = left bundle branch block; MI = myocardial infarction; CABG = coronary artery bypass graft; CAD = coronary artery disease; PCI = percutaneous coronary intervention; NSTE-ACS = non-ST-segment elevation acute coronary syndrome; MI = myocardial infarction; AMI = acute myocardial infarction; PPCI = primary percutaneous coronary intervention; NYHA = New York Heart Association functional classification; TIA = transient ischemic attack; GRACE = Global Registries of Acute Coronary Events; INR = international normalized ratio; Hb = hemoglobin; NSAIDs = non-steroidal anti-inflammatory drugs; COX = cyclooxygenase

Supplemental Table 4b. Baseline characteristics of included trials

| Trial | Arm | Age | Male | HT | DL | DM | Prior MI | Prior PCI | Prior CABG |
| --- | --- | --- | --- | --- | --- | --- | --- | --- | --- |
| TICAKORIA  Park et al. 2019 | Ticagrelor | 62.5 ± 11.3 | 74.2% | 55.8% | 52.0% | 29.0% | 6.2% | 10.2% | 1.0% |
|  | Clopidogrel | 62.3 ± 11.5 | 75.5% | 48.2% | 48.5% | 25.0% | 5.0% | 7.8% | 0.8% |
| ISAR REACT 5  Schupke et al. 2019 | Ticagrelor | 64.5 ± 12.0 | 76.2% | 71.3% | 58.7% | 23.0% | 15.5% | 22.5% | 5.7% |
|  | Prasugrel | 64.6 ± 12.1 | 76.2% | 69.1% | 58.1% | 24.1% | 16.0% | 23.1% | 6.5% |
| Elderly ACS 2  Savonitto et al. 2018 | Prasugrel | 80 (77–84)* | 59% | 78% | 47% | 30% | 19% | 20% | 8% |
|  | Clopidogrel | 80 (77–84)* | 61% | 78% | 43% | 28% | 19% | 16% | 10% |
| PRAGUE 18  Motovska et al. 2018 | Prasugrel | 61.8 (42.7–78.7) | 77.1% | 51.4% | 33.4% | 20.0% | 7.4% | 6.6% | 1.9% |
|  | Ticagrelor | 61.8 (44.6–79.8) | 73.7% | 51.2% | 35.4% | 20.8% | 9.2% | 7.6% | 1.5% |
| Tang et al. 2016 | Ticagrelor | 64.4 ± 11.4 | 71% | 61% | 44% | 29% | 8% | 100% | 0% |
|  | Clopidogrel | 64.2 ± 11.1 | 73% | 58% | 37% | 21% | 5% | 100% | 0% |
| PHILO  Goto et al. 2015 | Ticagrelor | 67 ± 12 | **76.3**% | 76.1% | 78.3% | 38.4% | 8.2% | 11.2% | 1.2% |
|  | Clopidogrel | 66 ± 11 | **76.7**% | 72.5% | 72.3% | 31.0% | 7.8% | 10.5% | 0.3% |
| PRASFIT-Elective  Isshiki et al. 2014 | Prasugrel | 67.5 ± 7.5 | 74.1% | 79.7% | 80.0% | 40.5% | 5.7% | NA | NA |
|  | Clopidogrel | 67.4 ± 7.4 | 70.7% | 81.7% | 82.0% | 35.5% | 4.3% | NA | NA |
| PRASFIT-ACS  Saito et al. 2014 | Prasugrel | 65.4 ± 11.4 | 78.2% | 7.23% | 75.3% | 36.5% | 5.0% | 5.8% | 0.9% |
|  | Clopidogrel | 65.1 ± 11.3 | 79.4% | 72.4% | 73.7% | 35.0% | 5.2% | 6.6% | 0.6% |
| TRILOGY ACS  Roe et al. 2012 | Prasugrel | 66 (58–74)* | 60.8% | 81.9% | 59.0% | 37.7% | 42.9% | 25.6% | 15.2% |
|  | Clopidogrel | 66 (59–73)* | 60.9% | 82.0% | 59.3% | 38.3% | 43.3% | 26.7% | 16.1% |
| PLATO  Wallentin et al. 2009 | Ticagrelor | 62.0* | 71.6% | 65.8% | 46.6% | 24.9% | 20.4% | 13.6% | 5.7% |
|  | Clopidogrel | 62.0* | 71.7% | 65.1% | 46.7% | 25.1% | 20.7% | 13.1% | 6.2% |
| Wang et al. 2016 | Ticagrelor | 79.0 (76–85)* | 69.0% | 79% | 84% | 42% | 17% | 3% | 0% |
|  | Clopidogrel | 80.0 (74–86)* | 66.0% | 82% | 79% | 39% | 15% | 6% | 0% |
| TRITON-TIMI 38  Wiviott et al. 2007 | Prasugrel | 61 (53–69)* | 75.0% | 64% | 56% | 23% | 18% | 99% | 1% |
|  | Clopidogrel | 61 (53–70)* | 73.0% | 64% | 56% | 23% | 18% | 99% | 1% |
| DISPERSE-2  Canon et al. 2007 | Ticagrelor | 64 ± 12.1 | 61% | NA | NA | 25% | 24% | 13% | 8% |
|  | Clopidogrel | 62 ± 11.0 | 66% | NA | NA | 25% | 28% | 17% | 11% |

HT = hypertension; DL = dyslipidemia; DM = diabetes mellitus; MI = myocardial infarction; PCI = percutaneous coronary intervention; CABG = coronary artery bypass graft

* Median

Supplemental Table 4c. Therapeutic interventions in individual trials

| Trial | Arm | Interventional strategy | Beta-blocker | Statin | ACEI/ARB |
| --- | --- | --- | --- | --- | --- |
| TICAKORIA  Park et al. 2019 | Ticagrelor | PCI 81.5%, CABG 2.8% | 68.8% | 88.5% | 40.8% |
|  | Clopidogrel | PCI 85.5%, CABG 1.5% | 74.2% | 92.2% | 42.8% |
| ISAR REACT 5  Schupke et al. 2019 | Ticagrelor | PCI 83.3% | 83.1% | 91.6% | 84.0% |
|  | Prasugrel | PCI 84.8% | 83.2% | 92.6% | 85.4% |
| Elderly ACS 2  Savonitto et al. 2018 | Prasugrel | PCI 100% | 78% | 95% | 83% |
|  | Clopidogrel | PCI 100% | 78% | 95% | 83% |
| PRAGUE 18  Motovska et al. 2018 | Prasugrel | PCI 100% | 82.3% | 93.8% | 84.2% |
|  | Ticagrelor | PCI 100% | 81.5% | 93.6% | 83.2% |
| Tang et al. 2016 | Ticagrelor | PCI 100% | 41% | 99% | 38% |
|  | Clopidogrel | PCI 100% | 48% | 99.5% | 47% |
| PHILO  Goto et al. 2015 | Ticagrelor | PCI 84.8%, CABG 2.2% | NA | NA | NA |
|  | Clopidogrel | PCI 84.5%, CABG 0.8% | NA | NA | NA |
| PRASFIT-Elective  Isshiki et al. 2014 | Prasugrel | PCI 100% | NA | NA | NA |
|  | Clopidogrel | PCI 100% | NA | NA | NA |
| PRASFIT-ACS  Saito et al. 2014 | Prasugrel | PCI 100% | NA | NA | NA |
|  | Clopidogrel | PCI 100% | NA | NA | NA |
| TRILOGY ACS  Roe et al. 2012 | Prasugrel | NA | NA | NA | NA |
|  | Clopidogrel | NA | NA | NA | NA |
| PLATO  Wallentin et al. 2009 | Ticagrelor | PCI 60.9%, cardiac surgery 4.3% | 89.3% | 89.7% | 88.2% |
|  | Clopidogrel | PCI 61.1%, cardiac surgery 4.7% | 89.7% | 89.2% | 87.1% |
| Wang et al. 2016 | Ticagrelor | PCI 75%, CABG 0% | 69% | 83% | 61% |
|  | Clopidogrel | PCI 71%, CABG 0% | 74% | 79% | 67% |
| TRITON-TIMI 38  Wiviott et al. 2007 | Prasugrel | PCI 99%, CABG 1% | NA | NA | NA |
|  | Clopidogrel | PCI 99%, CABG 1% | NA | NA | NA |
| DISPERSE-2  Canon et al. 2007 | Ticagrelor | NA | NA | NA | NA |
|  | Clopidogrel | NA | NA | NA | NA |

ACEI = angiotensin-converting-enzyme inhibitor; ARB = angiotensin II receptor blockers; PCI = percutaneous coronary intervention ; CABG = coronary artery bypass graft

Supplemental Table 5a. League table for MACE

| Clopidogrel | 0.73 (0.49–1.09) | 0.86 (0.68–1.09) | 1.02 (0.62–1.67) |
| --- | --- | --- | --- |
| 1.37 (0.92–2.05) | Prasugrel (low dose) | 1.18 (0.74–1.88) | 1.30 (0.81–2.08) |
| 1.16 (0.92–1.48) | 0.85 (0.53–1.35) | Prasugrel (standard dose) | 1.10 (0.78–1.55) |
| 0.98 (0.60–1.62) | 0.77 (0.48–1.24) | 0.91 (0.64–1.28) | Ticagrelor |

Supplemental Table 5b. League table for bleeding events

| Clopidogrel | 0.72 (0.35–1.49) | 1.26 (1.01–1.58) | 1.26 (0.82–1.96) |
| --- | --- | --- | --- |
| 1.39 (0.67–2.87) | Prasugrel (low dose) | 1.75 (0.82–3.74) | 1.53 (0.70–3.34) |
| 0.79 (0.63–0.99) | 0.57 (0.27–1.22) | Prasugrel (standard dose) | 0.87 (0.61–1.25) |
| 0.79 (0.51–1.23) | 0.66 (0.30–1.43) | 1.15 (0.80–1.65) | Ticagrelor |

Supplemental Table 5c. League table for CV death

| Clopidogrel | 1.45 (0.54–3.91) | 0.92 (0.81–1.03) | 1.06 (0.76–1.48) |
| --- | --- | --- | --- |
| 0.69 (0.26–1.87) | Prasugrel (low dose) | 0.63 (0.23–1.72) | 0.61 (0.23–1.66) |
| 1.09 (0.97–1.23) | 1.58 (0.58–4.31) | Prasugrel (standard dose) | 0.97 (0.84–1.11) |
| 0.94 (0.67–1.32) | 1.64 (0.60–4.43) | 1.03 (0.90–1.19) | Ticagrelor |

Supplemental Table 5d. League table for MI

| Clopidogrel | 0.81 (0.54–1.22) | 0.84  (0.67–1.06) | 1.27 (0.74–2.16) |
| --- | --- | --- | --- |
| 1.24 (0.82–1.86) | Prasugrel (low dose) | 1.04 (0.65–1.67) | 1.11 (0.69–1.79) |
| 1.18 (0.95–1.48) | 0.96 (0.60–1.53) | Prasugrel (standard dose) | 1.06 (0.75–1.49) |
| 0.79 (0.46–1.34) | 0.90 (0.56–1.45) | 0.94 (0.67–1.32) | Ticagrelor |

Supplemental Table 5e. League table for stroke

| Clopidogrel | 0.67 (0.23–1.97) | 0.92 (0.72–1.16) | 0.80 (0.42–1.53) |
| --- | --- | --- | --- |
| 1.49 (0.51–4.36) | Prasugrel (low dose) | 1.36 (0.45–4.10) | 1.72 (0.57–5.18) |
| 1.09 (0.86–1.38) | 0.73 (0.24–2.21) | Prasugrel (standard dose) | 1.26 (0.90–1.77) |
| 1.24 (0.65–2.36) | 0.58 (0.19–1.75) | 0.79 (0.57–1.11) | Ticagrelor |

Supplemental Table 6. Local inconsistency test between direct and indirect treatment comparisons in mixed treatment comparison

| Comparison | Direct | | Indirect | | Difference | | | Tau |
| --- | --- | --- | --- | --- | --- | --- | --- | --- |
|  | Coefficient | Std. error | Coefficient | Std. error | Coefficient | Std. error | P-value |  |
| C - P (S) | -0.152 | 0.122 | -0.168 | 0.221 | 0.016 | 0.255 | 0.95 | 0.186 |
| C - T | -0.057 | 0.125 | -0.041 | 0.219 | -0.016 | 0.255 | 0.95 | 0.186 |
| P (S) - T | 0.111 | 0.179 | 0.095 | 0.176 | 0.016 | 0.255 | 0.95 | 0.186 |

C = clopidogrel; P (L) = prasugrel (low dose); P (S) = prasugrel (standard dose); T = ticagrelor

Supplemental Figure 1a. Cochrane risk of bias assessment


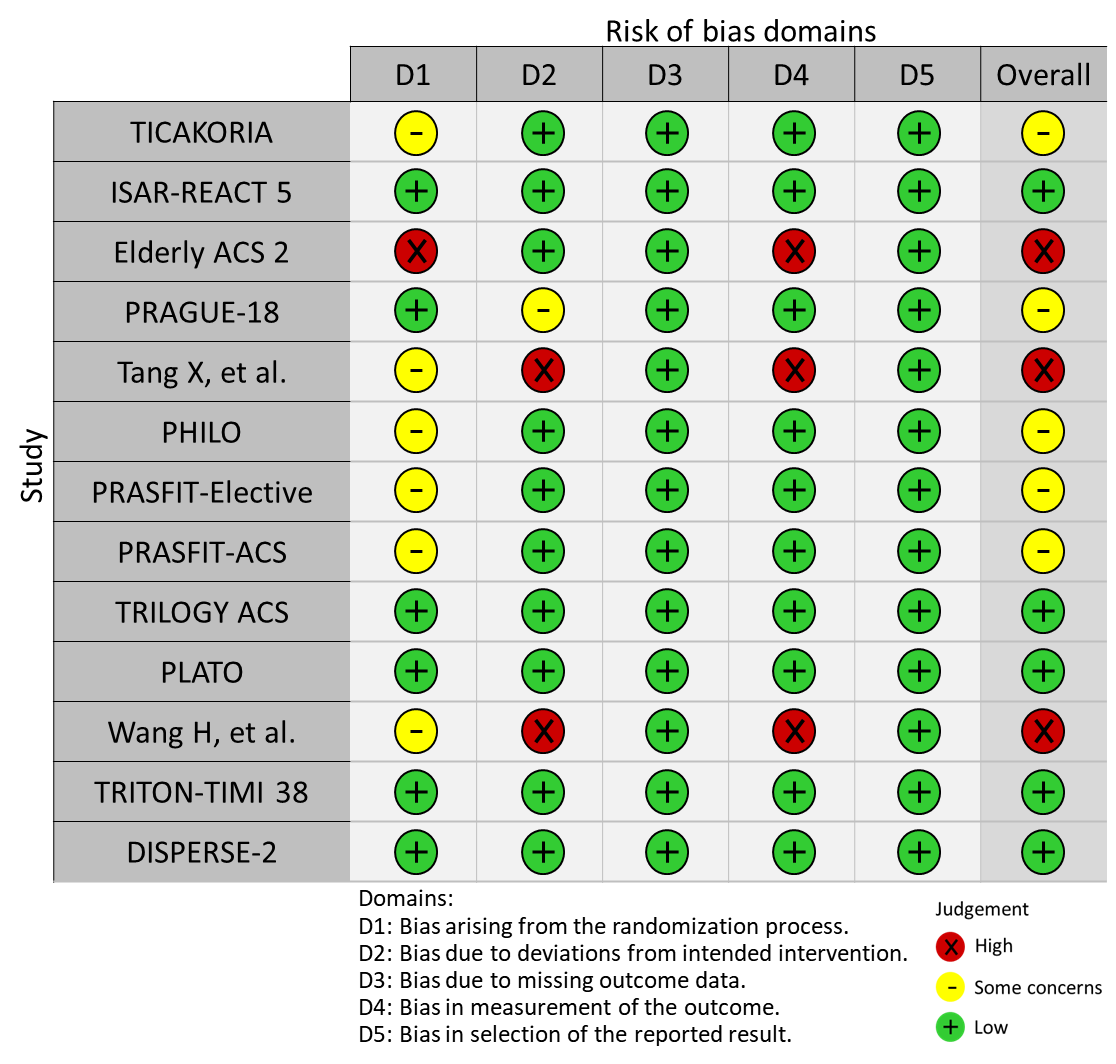


Supplemental Figure 1b. Cochrane risk of bias graph presented as percentages
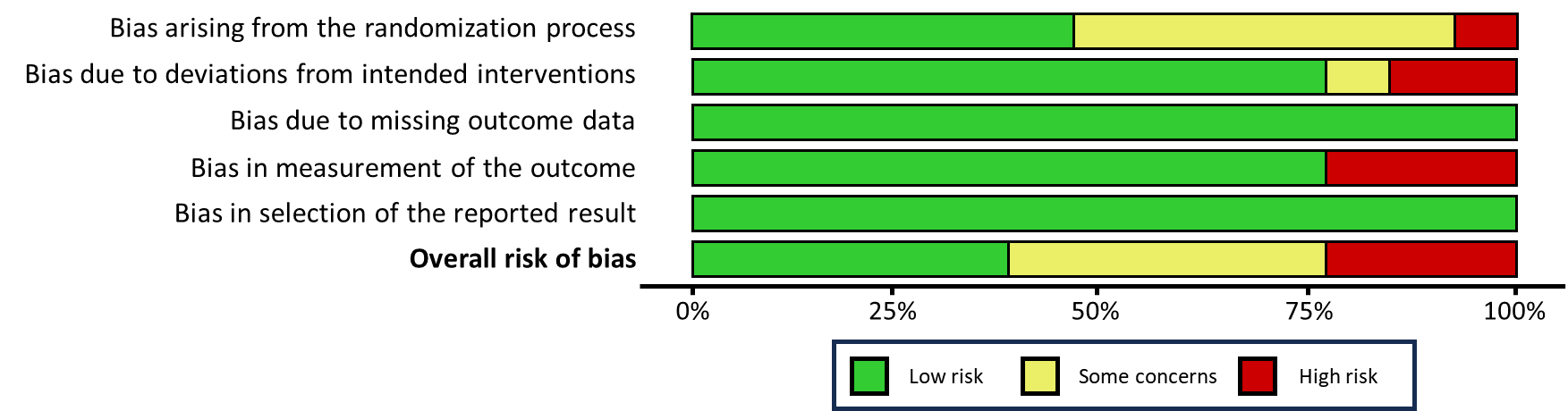


Supplemental Figure 2a. Direct pairwise meta-analysis of randomized trials for CV death
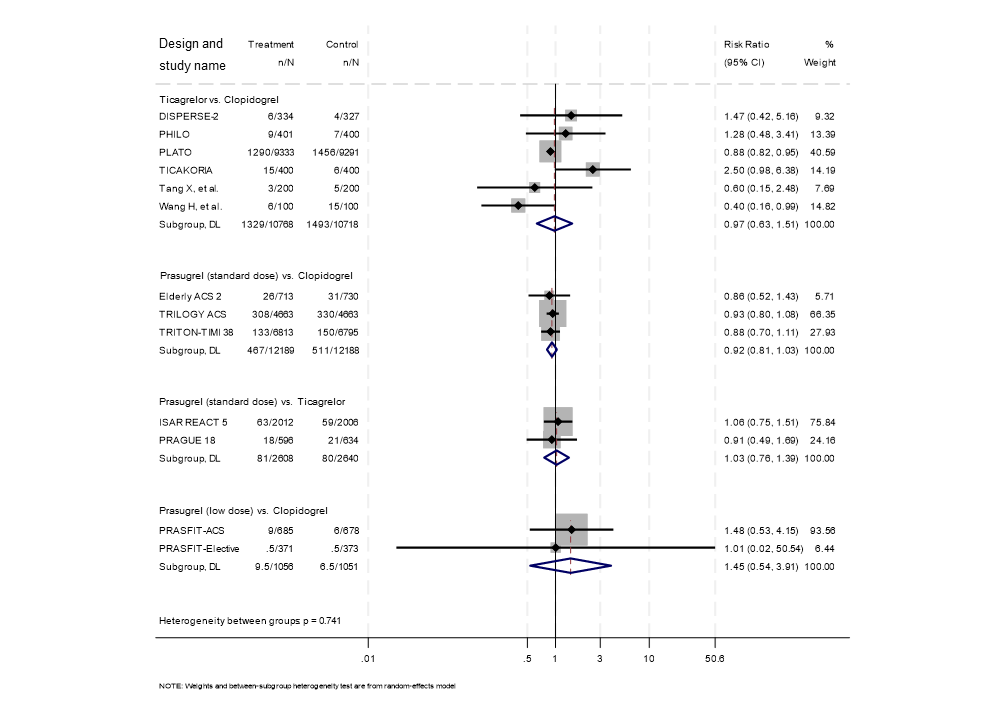


Supplemental Figure 2b. Direct pairwise meta-analysis of randomized trials for MI
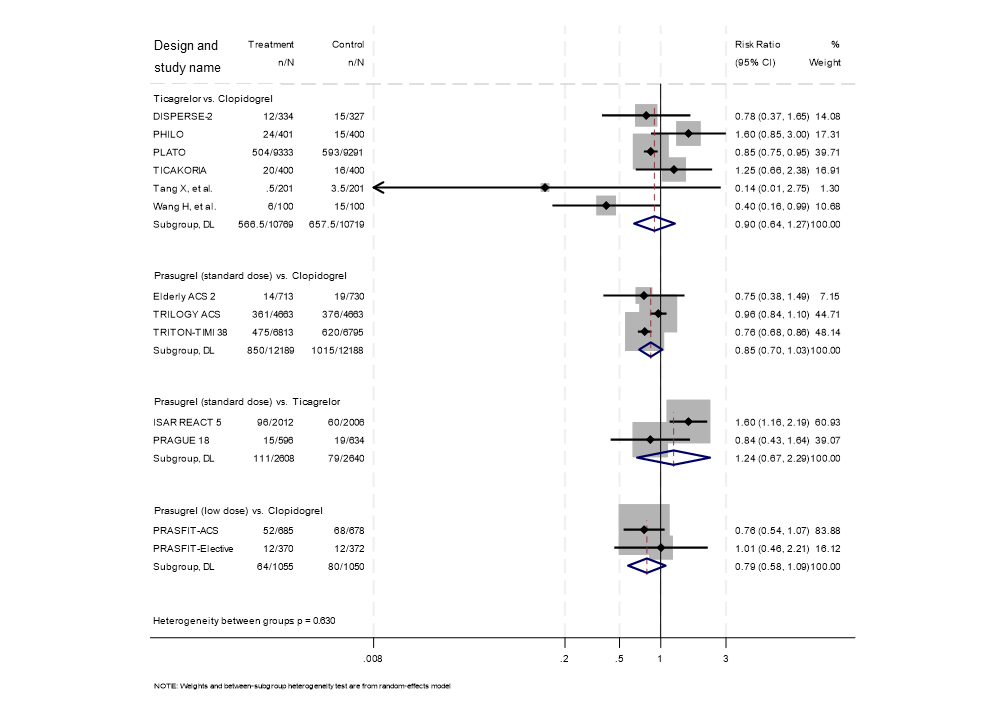


Supplemental Figure 2c. Direct pairwise meta-analysis of randomized trials for stroke


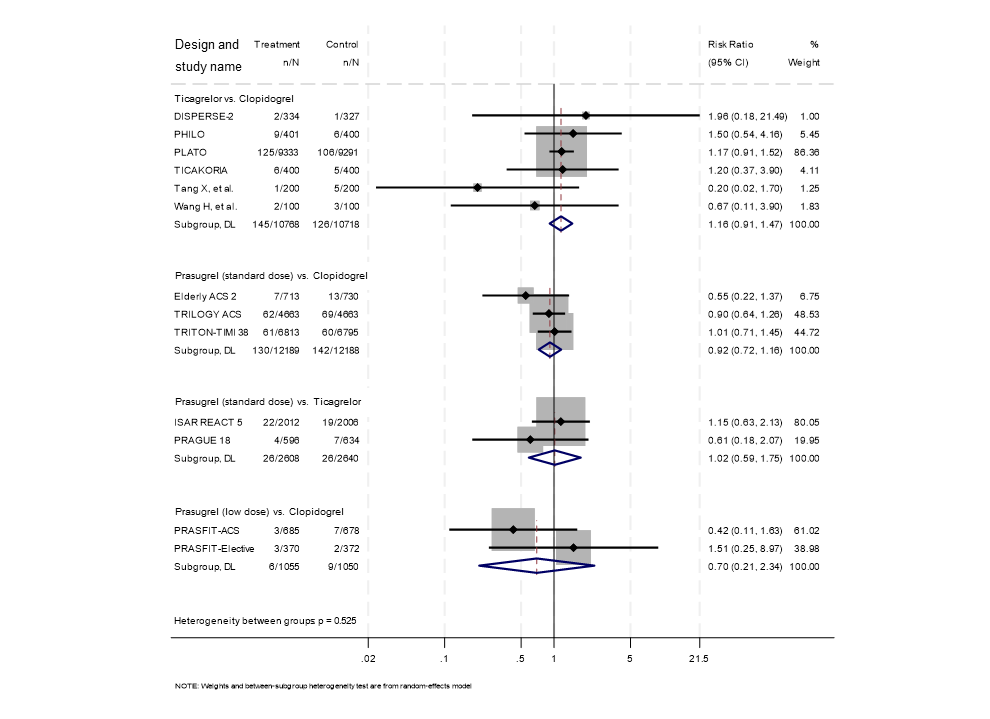


Supplemental Figure 3. Contribution plot


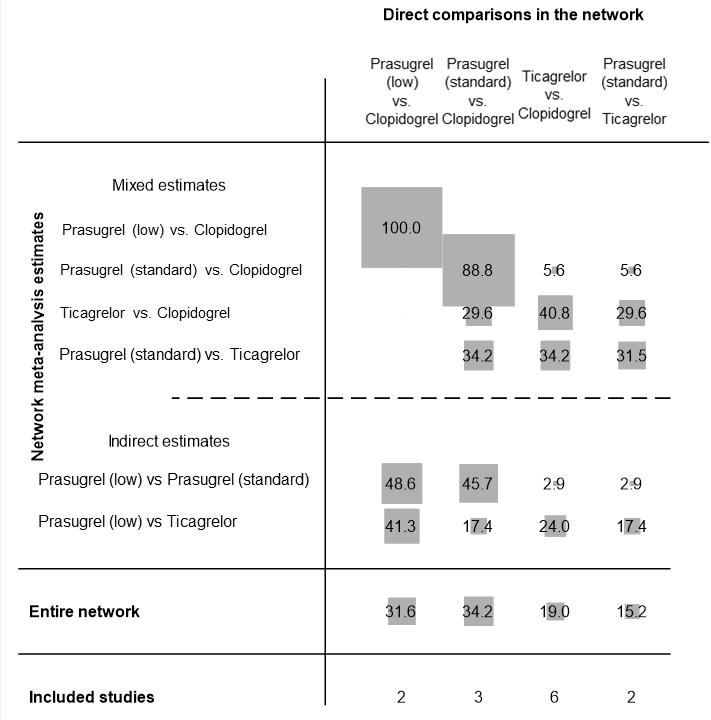


Supplemental Figure 4. Forest plot of network meta-analysis for MACE
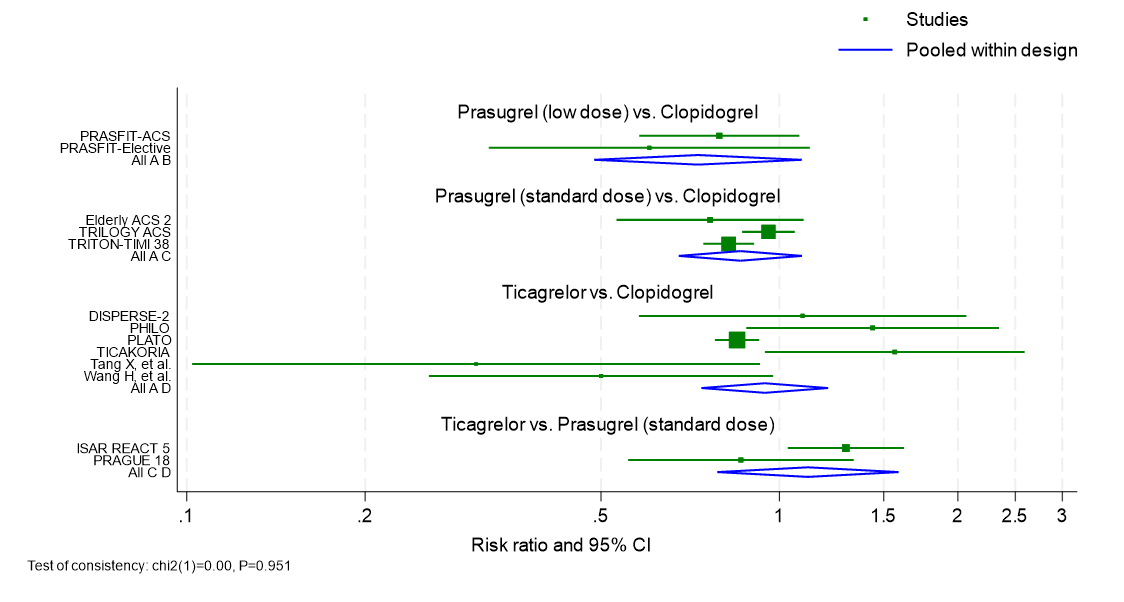


Supplemental Figure 5. Forest plot of network meta-analysis for bleeding events
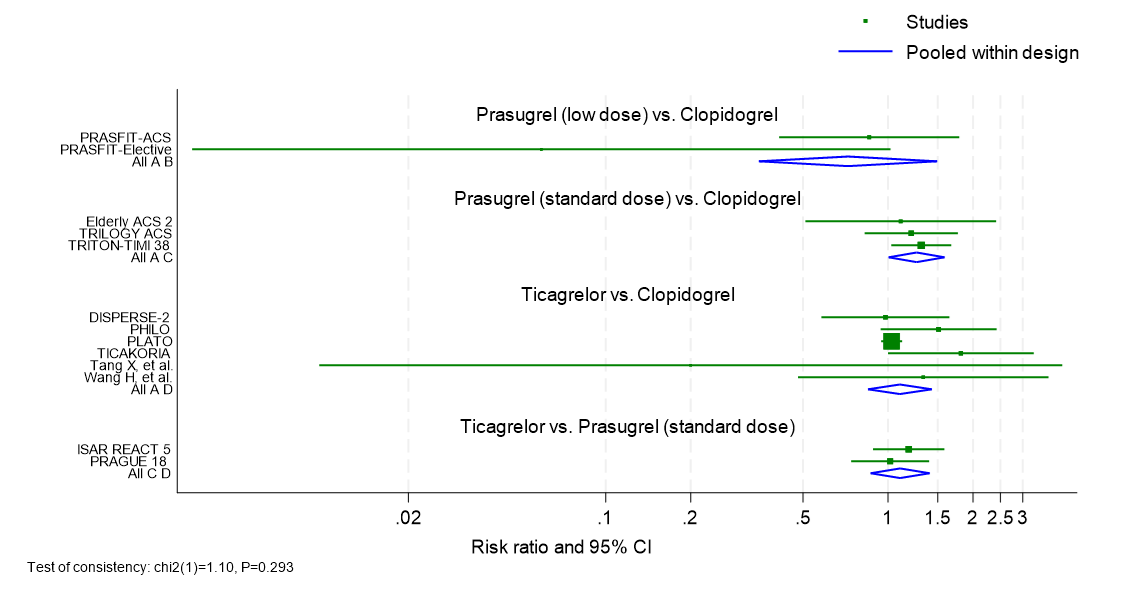


Supplemental Figure 6. Network meta-analysis for CV death
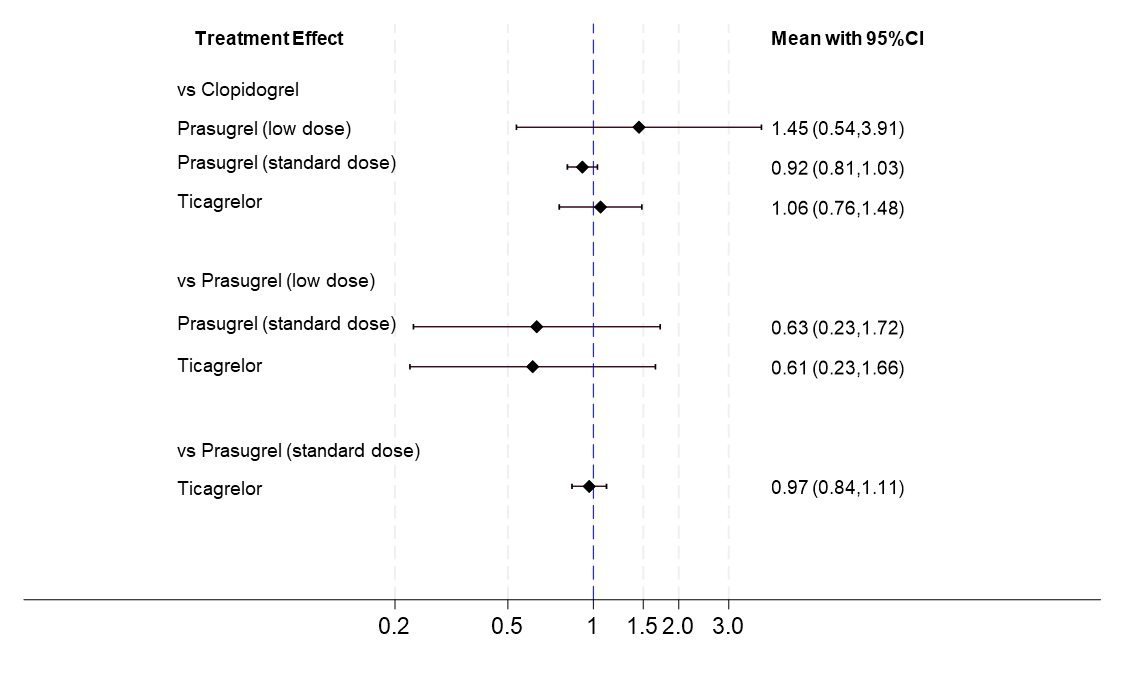


Supplemental Figure 7. Forest plot of network meta-analysis for CV death
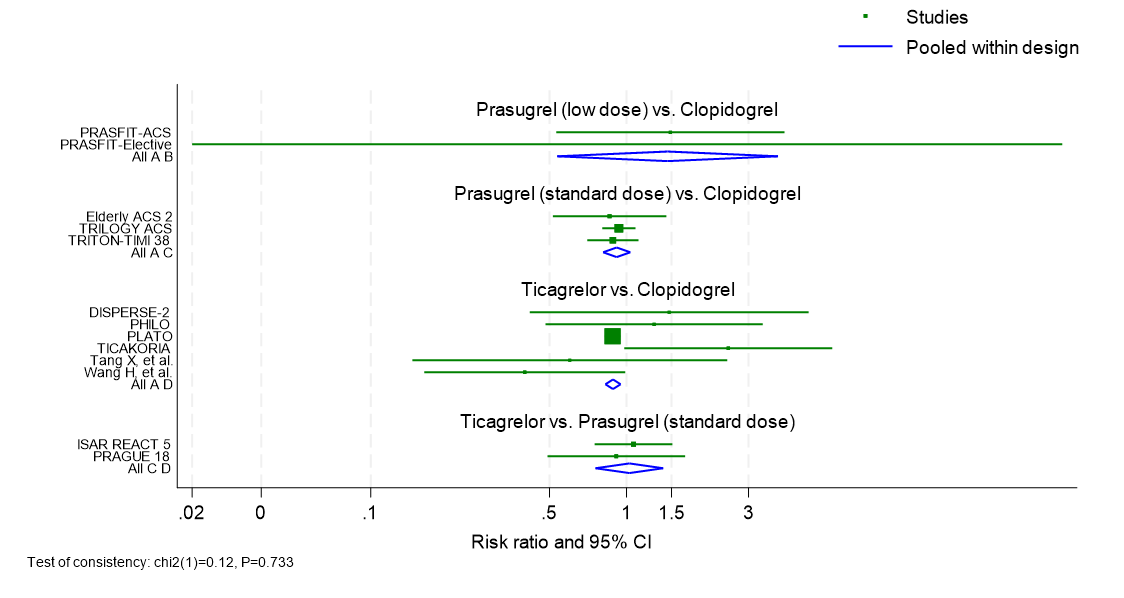


Supplemental Figure 8. Network meta-analysis for MI
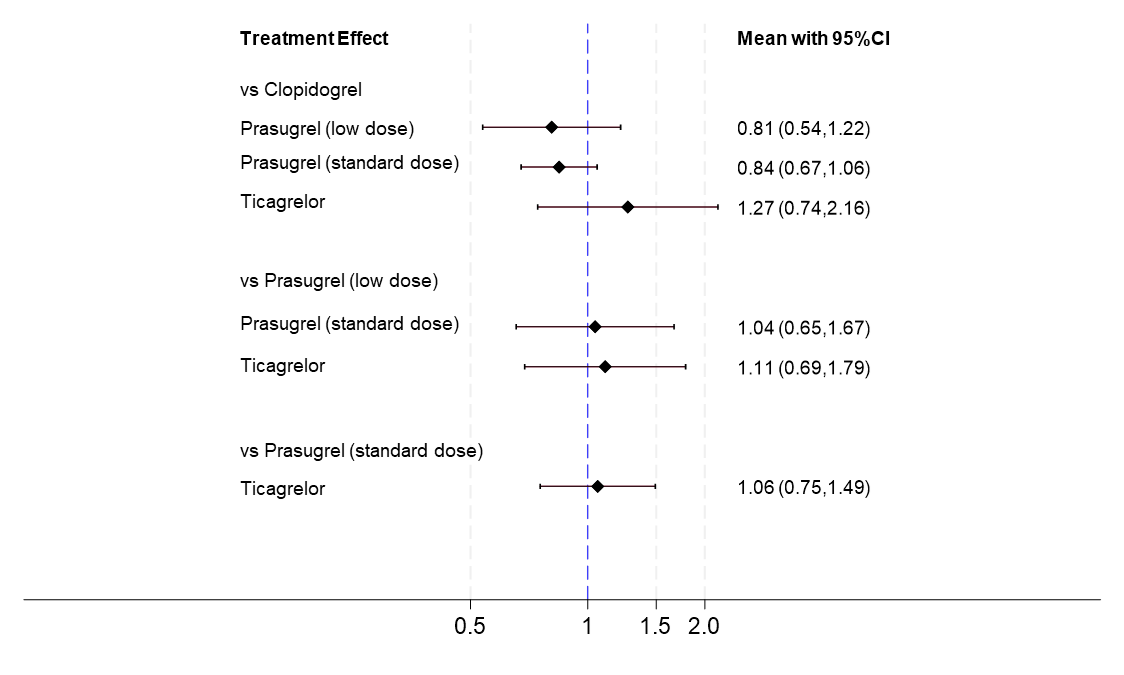


Supplemental Figure 9. Forest plot of network meta-analysis for MI
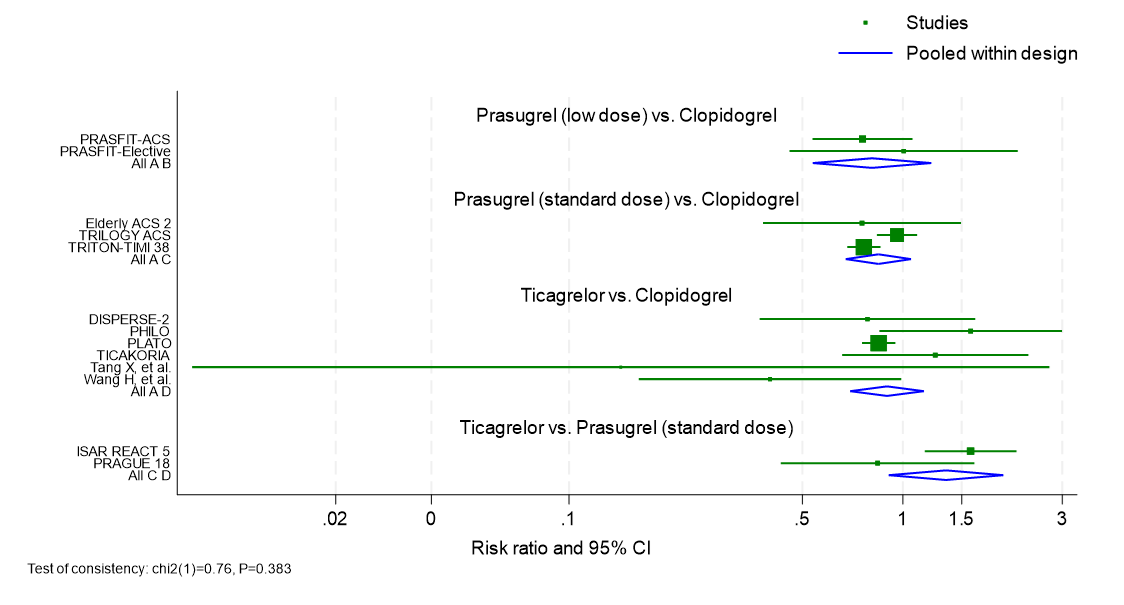


Supplemental Figure 10. Network meta-analysis for stroke
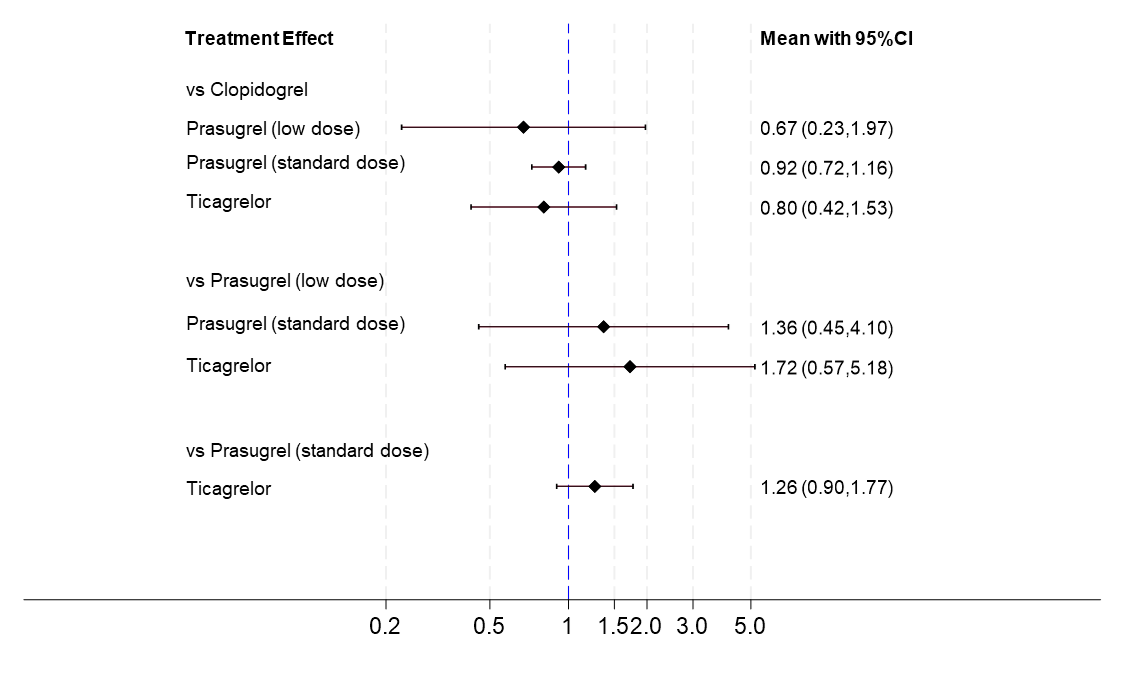


Supplemental Figure 11. Forest plot of network meta-analysis for stroke
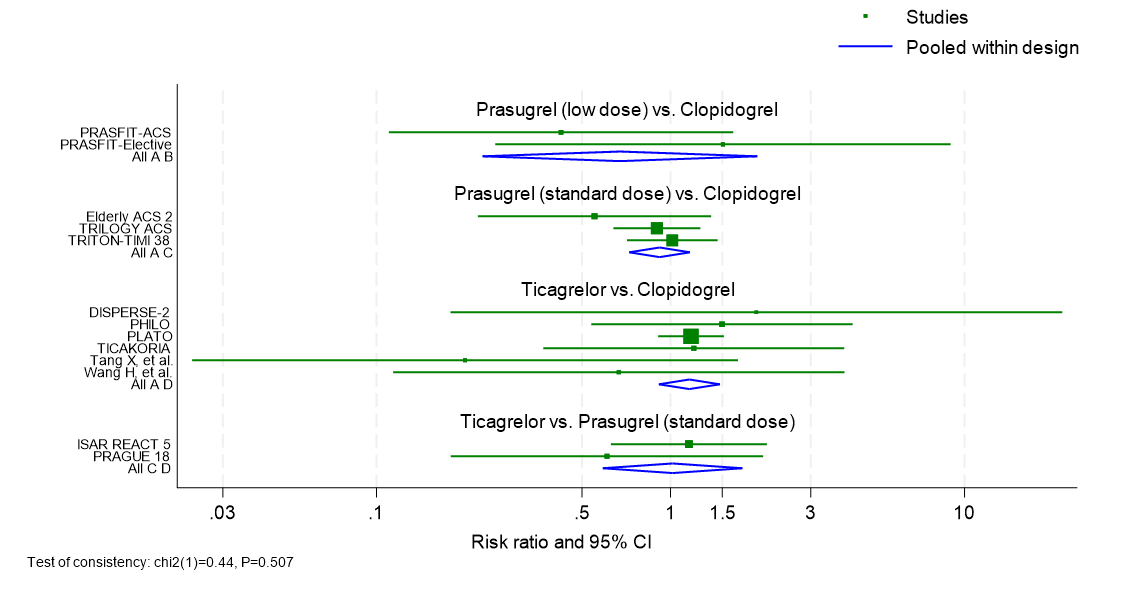


Supplemental Figure 12a. Sensitivity analysis: Excluding trials with a high risk of bias, MACE
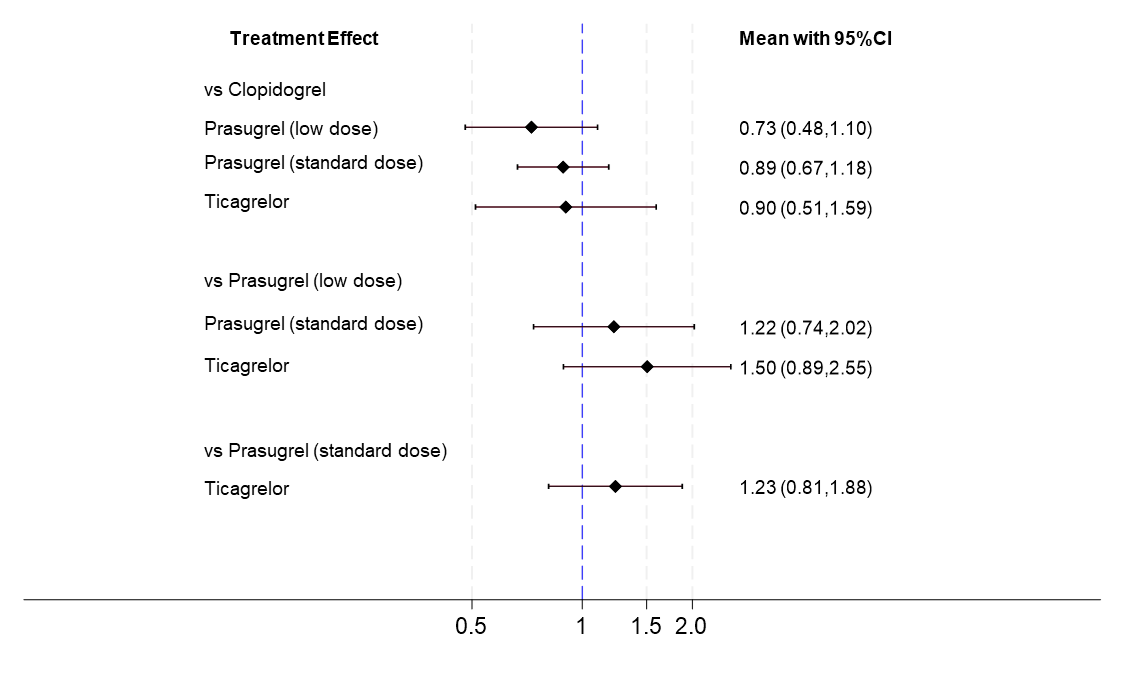


Supplemental Figure 12b. Sensitivity analysis: Excluding trials with a high risk of bias, bleeding events
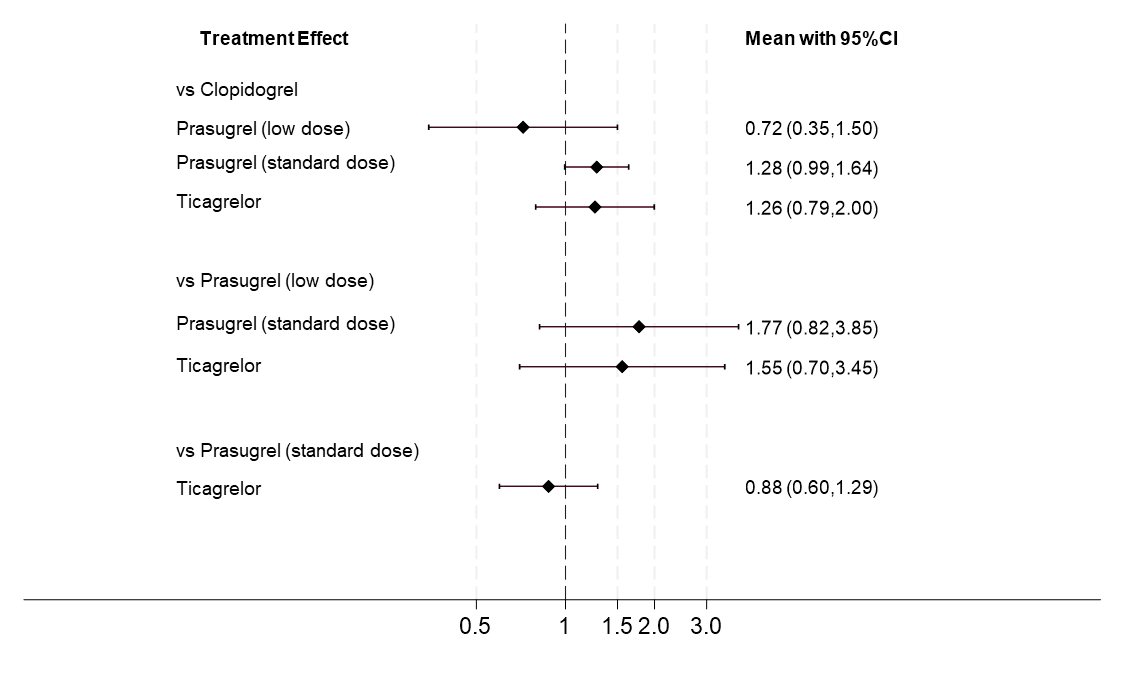


Supplemental Figure 12c. Sensitivity analysis: Excluding trials with a high risk of bias, CV death
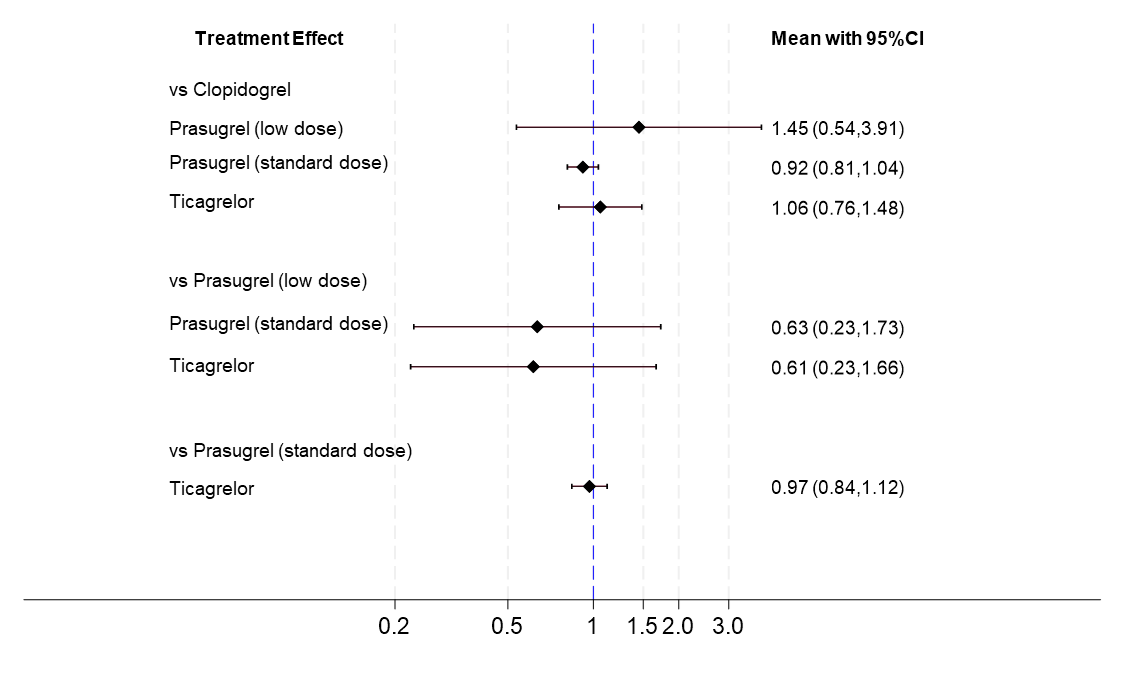


Supplemental Figure 12d. Sensitivity analysis: Excluding trials with a high risk of bias, MI
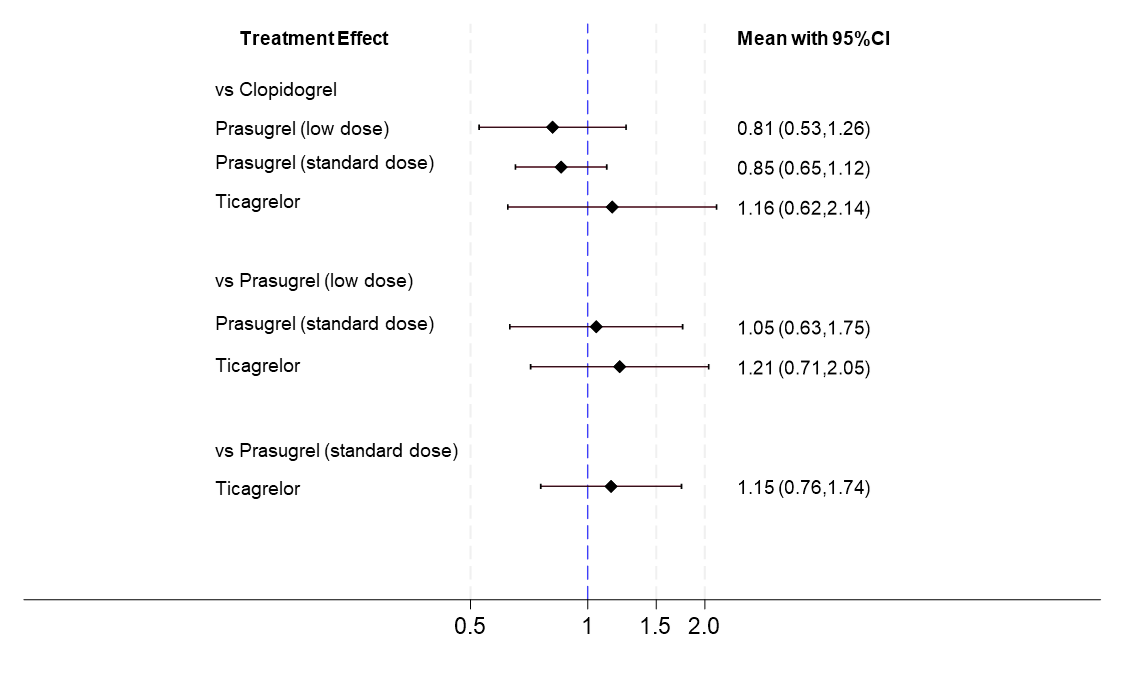


Supplemental Figure 12e. Sensitivity analysis: Excluding trials with a high risk of bias, stroke
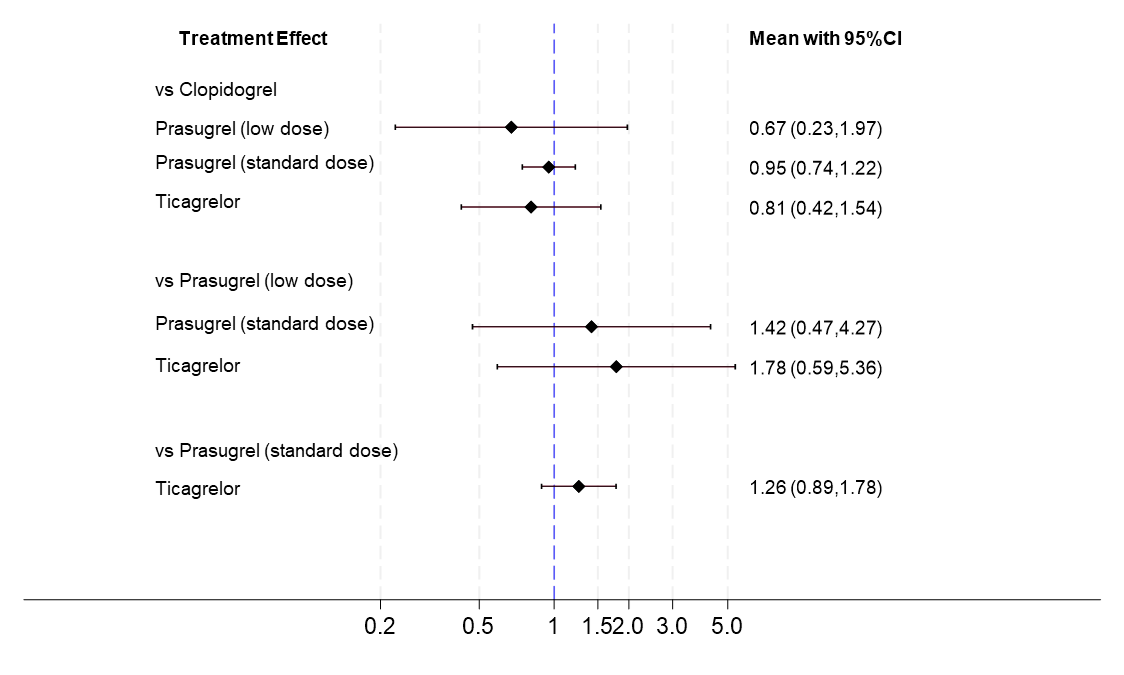


Supplemental Figure 13a. Sensitivity analysis: Excluding small-sample trials, MACE
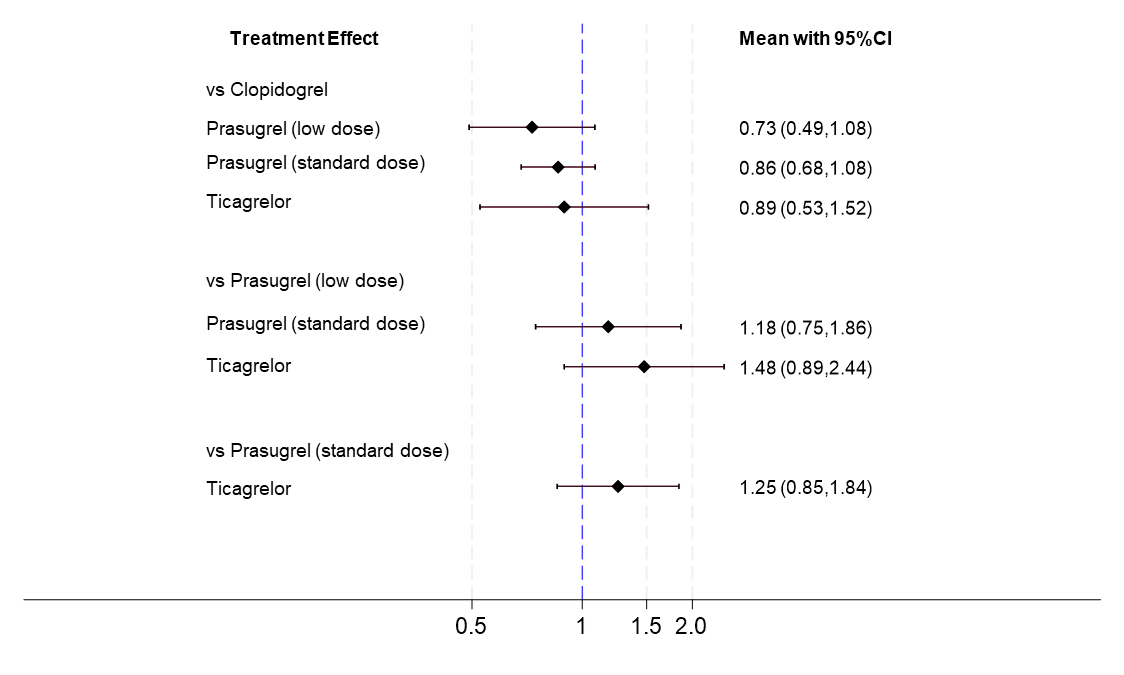


Supplemental Figure 13b. Sensitivity analysis: Excluding small-sample trials, bleeding events
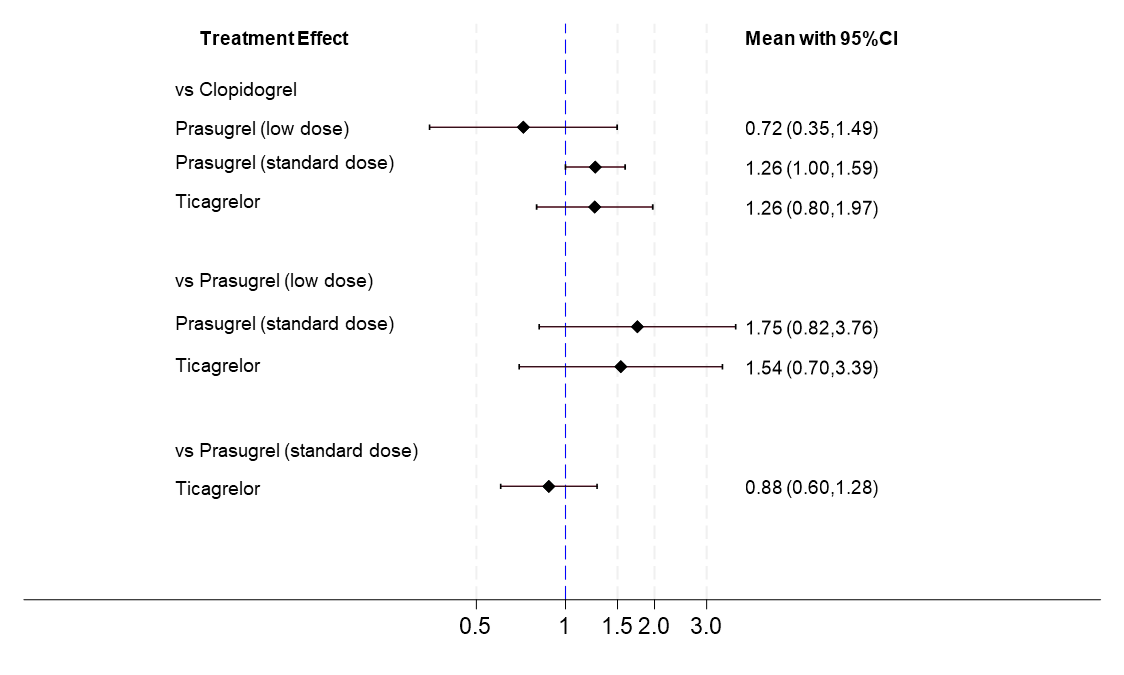


Supplemental Figure 13c. Sensitivity analysis: Excluding small-sample trials, CV death
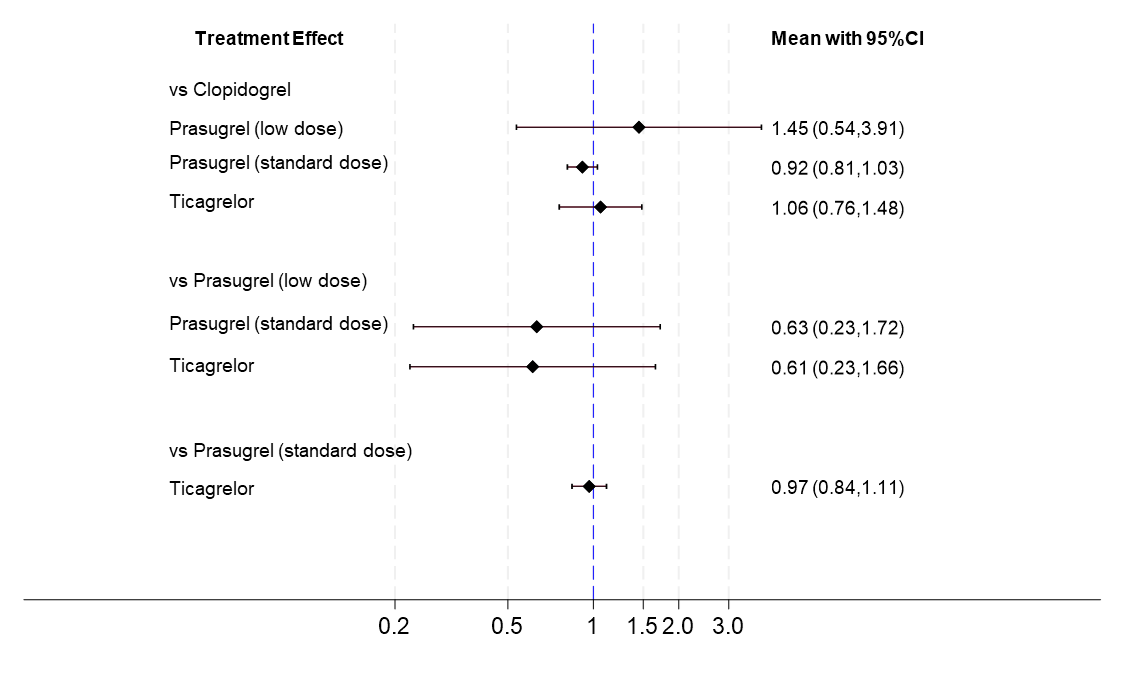


Supplemental Figure 13d. Sensitivity analysis: Excluding small-sample trials, MI
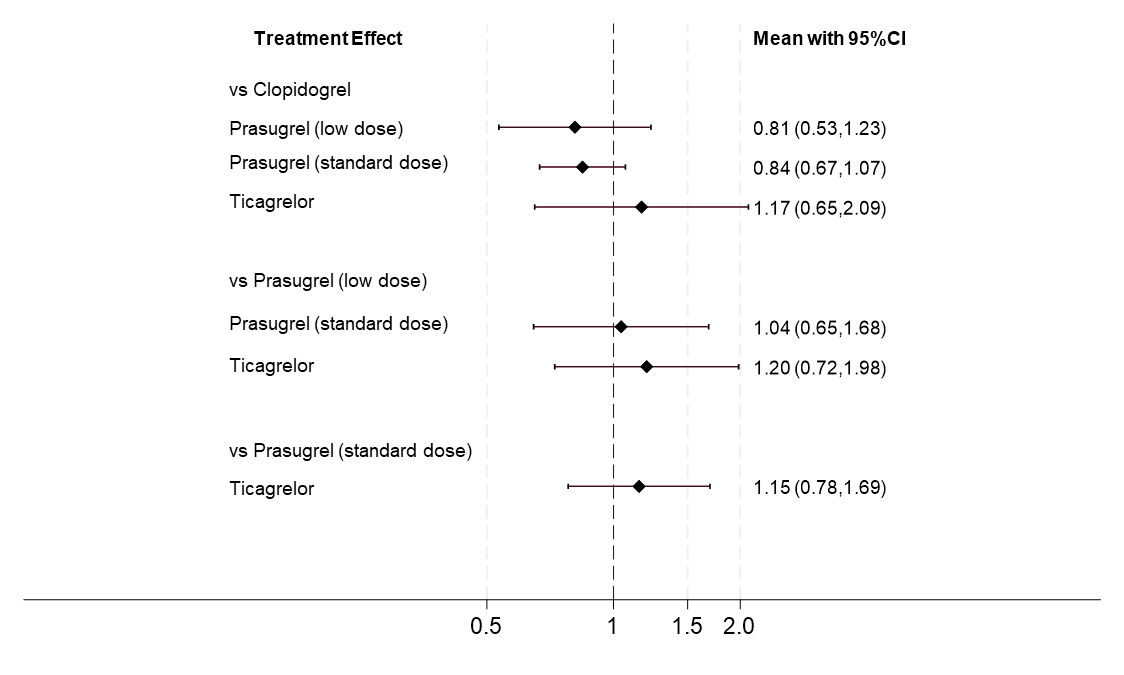


Supplemental Figure 13e. Sensitivity analysis: Excluding small-sample trials, stroke
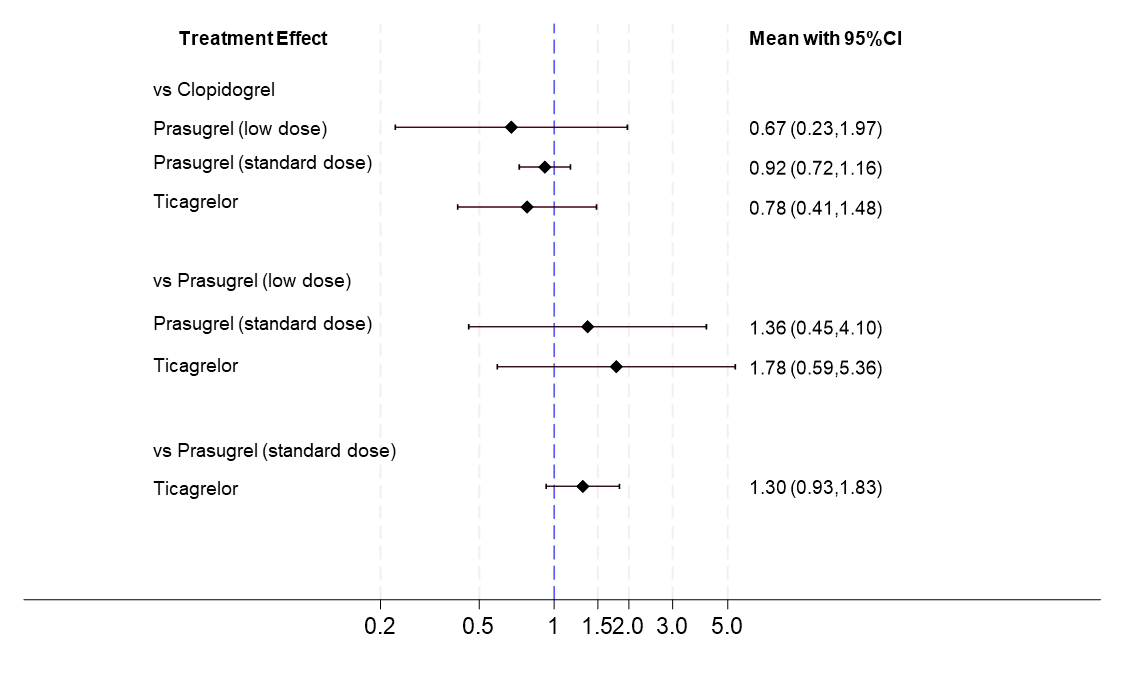


Supplemental Figure 14a. Funnel plot for MACE


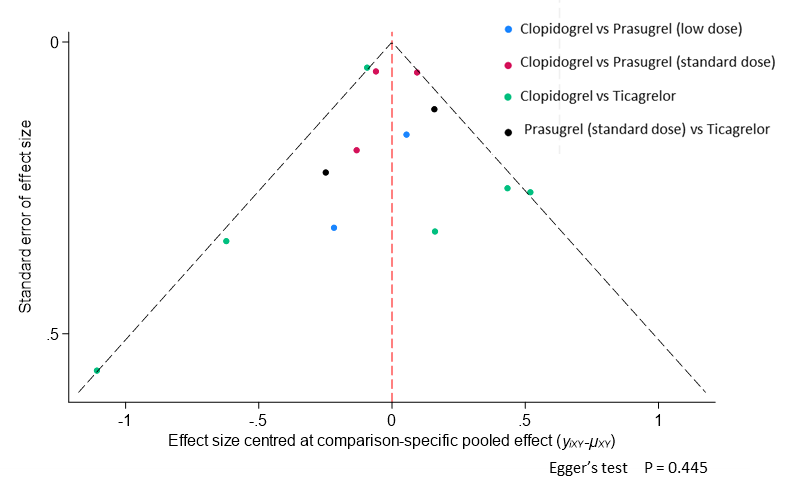


Supplemental Figure 14b. Funnel plot for bleeding events


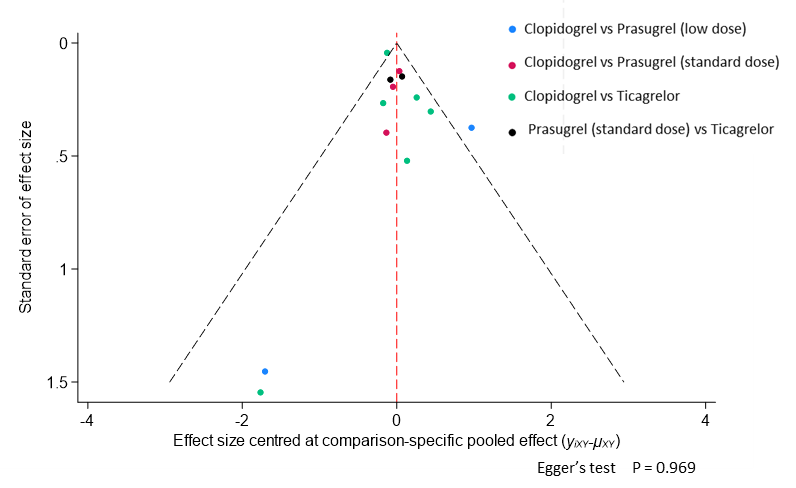

Supplement: Supplementary file 1 — Supplementary file1 (DOCX 1287 KB) [file 12928_2025_1129_MOESM1_ESM.docx]
